# Expression of Eukaryotic Initiation Factor 5A and Hypusine Forming Enzymes in Glioblastoma Patient Samples: Implications for New Targeted Therapies

**DOI:** 10.1371/journal.pone.0043468

**Published:** 2012-08-21

**Authors:** Michael Preukschas, Christian Hagel, Alexander Schulte, Kristoffer Weber, Katrin Lamszus, Henning Sievert, Nora Pällmann, Carsten Bokemeyer, Joachim Hauber, Melanie Braig, Stefan Balabanov

**Affiliations:** 1 Department of Oncology, Haematology and Bone Marrow Transplantation with Section Pneumology, Hubertus Wald-Tumorzentrum, University Medical Center Hamburg-Eppendorf, Hamburg, Germany; 2 Department of Neuropathology, University Medical Center Hamburg-Eppendorf, Hamburg, Germany; 3 Department of Neurosurgery, University Medical Center Hamburg-Eppendorf, Hamburg, Germany; 4 Research Department Cell and Gene Therapy, Clinic for Stem Cell Transplantation, University Medical Center Hamburg-Eppendorf, Hamburg, Germany; 5 Heinrich-Pette-Institute – Leibniz Institute for Experimental Virology, Hamburg, Germany; City of Hope National Medical Center and Beckman Research Institute, United States of America

## Abstract

Glioblastomas are highly aggressive brain tumors of adults with poor clinical outcome. Despite a broad range of new and more specific treatment strategies, therapy of glioblastomas remains challenging and tumors relapse in all cases. Recent work demonstrated that the posttranslational hypusine modification of the eukaryotic initiation factor 5A (eIF-5A) is a crucial regulator of cell proliferation, differentiation and an important factor in tumor formation, progression and maintenance. Here we report that eIF-5A as well as the hypusine-forming enzymes deoxyhypusine synthase (DHS) and deoxyhypusine hydroxylase (DOHH) are highly overexpressed in glioblastoma patient samples. Importantly, targeting eIF-5A and its hypusine modification with GC7, a specific DHS-inhibitor, showed a strong antiproliferative effect in glioblastoma cell lines *in vitro*, while normal human astrocytes were not affected. Furthermore, we identified p53 dependent premature senescence, a permanent cell cycle arrest, as the primary outcome in U87-MG cells after treatment with GC7. Strikingly, combined treatment with clinically relevant alkylating agents and GC7 had an additive antiproliferative effect in glioblastoma cell lines. In addition, stable knockdown of eIF-5A and DHS by short hairpin RNA (shRNA) could mimic the antiproliferative effects of GC7. These findings suggest that pharmacological inhibition of eIF-5A may represent a novel concept to treat glioblastomas and may help to substantially improve the clinical course of this tumor entity.

## Introduction

Gliomas are the most frequent primary brain tumors in adults. As a result of diffuse infiltration, surgical resection of glioblastomas (GBM) is difficult and the tumor usually relapses within months. Adjuvant radiochemotherapy with alkylating agents like temozolomide (TMZ), followed by cyclic TMZ therapy is able to delay tumor progression and helps to increase the overall survival up to 14–15 months [1], [2]. However, tumors become resistant to these therapies e.g. by alterations in growth and survival signalling pathways [3] or expression of DNA repair enzymes [4]. Based on these observations, an extraordinary effort has been made to identify novel therapeutic targets in GBM. In this context a comprehensive list of tumor specific alterations were encountered using new sequencing technologies [5], [6]. However, targeted therapies directed against growth factor receptors, downstream regulatory pathways or angiogenic capabilities of tumors by tyrosine kinase inhibitors or monoclonal antibodies lacked efficacy so far [7]–[10]. The disappointing results of current targeted therapies in GBM and the frequent emergence of resistance illustrates the urgent need for potential new molecular targets, which would ideally be independent of the above-mentioned mechanisms.

Here we present the highly specific posttranslational hypusine modification of the eukaryotic initiation factor 5A (eIF-5A) as a potential new target in glioblastoma. eIF-5A is a small acidic protein carrying a unique post-translational modification termed hypusine [11], [12]. The synthesis of this unusual amino acid (a spermidine dependent process termed hypusination) is carried out in two subsequent steps by the enzymes deoxyhypusine synthase (DHS) and deoxyhypusine hydroxylase (DOHH) at a conserved lysine residue of eIF-5A and leads to an activation of the protein [13]. In humans, two isofoms of eIF-5A, eIF-5A1 and eIF-5A2, have been described [14], [15]. Both isoforms have a high degree of amino acid homology but a different pattern of tissue expression. eIF-5A1 is ubiquitously expressed, whereas eIF-5A2 has been detected mainly in the testis and in the brain [15]. Noteworthy, the hypusine modification of eIF-5A is highly conserved in all eukaryotes and in some archaea and seems to be essential for the viability of eukaryotic cells [16]. The assumption is based on the observation that an inactivation of the hypusine formation is lethal for yeast, mice and eukaryotic cell lines [17]–[19]. Although eIF-5A was initially described as a translation initiation factor, more recent studies have highlighted that eIF-5A has additional cellular functions, including mRNA transport, binding of HIV REV and HTLV-I REX transactivator proteins as well as regulation of mRNA turnover [20], [21]. Besides the well established role of eIF-5A in proliferation and survival of eukaryotic cells, an involvement in the regulation of apoptosis was brought into focus (see [22], [23] for comprehensive reviews). An role of hypusine modification in the induction of apoptosis has been indicated by Tome and Gerner [24], [25]. They showed, that an excess accumulation of the polyamine putrescine leads to a reduced formation of hypusinated eIF-5A and apoptosis in DH23A/b hepatoma cells. Caraglia *et al.* identified interferon-α (IFNα) as a strong inducer of growth inhibition/apopstosis in human epidermoid cancer KB cells. This observation was accompanied by a strong inhibition of hypusine synthesis [26]. Interestingly, the combination of IFNα and the DHS inhibitor GC7 had a synergistic effect on the induction of cell growth inhibition and apoptosis in those cells [27]. In our recent work we found eIF-5A to be overexpressed in chronic myeloid leukemia patients and co-treatment of *BCR-ABL^+^* cells with imatinib and inhibitors of hypusine synthesis yielded a synergistic effect [28]. Further, eIF-5A and eIF-5A2 have already been associated with several other malignancies in the past. eIF-5A was found to be overexpressed in samples from colorectal adenoma and eIF-5A2 is present in various cancer cell lines and its overexpression may serve as a prognostic marker in patients with urothelial carcinoma or ovarian cancer [29]–[31]. Additionally, eIF-5A and/or eIF-5A2 have been proposed as a transforming and predictive factor in the development of hepatocellular carcinoma, non-small cell lung cancer and in patients with ovarian carcinoma [32]–[34]. Recently, Lu et al. reported that an ectopic expression of microRNA-7 leads to a downregulation of eIF-5A and reduced cell migration, invasion, and tumorigenesis in a glioma model [35]. Thus we investigated the potential of eIF-5A and the hypusine forming enzymes as possible novel targets for glioblastoma therapy.

We evaluated protein expression levels of eIF-5A1/2, DHS and DOHH in 173 glioma tumor samples of different grades as well in cell lines and analyzed the effect of inhibition of hypusination on glioblastoma cells *in vitro*. We investigated the proliferation, apoptosis and cell cycle in hypusination-impaired cells. Further, we explored the effect of clinically used alkylating agents on hypusine formation and potential additive antiproliferative effects in combination with DHS inhibition.

## Materials and Methods

### Reagents

N1-Guanyl-1,7-diaminoheptane (GC7) was purchased from Biosearch Technologies and was dissolved in sterile, millipore filtered H_2_O to a final concentration of 10 mM. Bis-chloroethylnitrosourea (BCNU, also known as Carmustine) and Temolozomide (TMZ) were obtained from Sigma-Aldrich. BCNU was dissolved in 50% EtOH at a concentration of 10 mM, whereas TMZ was dissolved in DMSO at a concentration of 50 mM.

### Cell Culture

Glioblastoma cell line G55T2 was already established from primary tumour material previously, as described in [36], [37]. U87-MG cells were obtained from American Type Culture Collection (Manassas, USA). Cell lines were maintained in Dulbecco’s modified Eagle medium (DMEM)-high glucose + Glutamax™ supplemented with 10% FBS, 1 mM sodium pyruvate and penicillin (50 U/ml)/streptomycin (50 µg/ml) (all from Invitrogen). Cells were passaged every 2–3 days. Because of their very high proliferation rate, G55T2 cells were grown in said medium with 5% FBS prior to cell cycle analysis. Primary normal human astrocytes (NHA) were purchased from Invitrogen and cultivated in DMEM-high glucose + Glutamax™ supplemented with 20% FBS, 1 mM sodium pyruvate and penicillin-streptomycin. All cell lines were cultivated at 37°C in 100% humidity and 5% CO_2_ and tested every few month for mycoplasma contamination.

### Immunohistochemistry

Immunohistochemical detection of eIF-5A, eIF-5A2, DHS and DOHH was performed on 4 µm sections of a tissue microarray (TMA) and samples of normal human brain. The TMA comprised 30 pilocytic astrocytomas WHO grade I, 30 diffuse astrocytomas WHO grade II, 27 anaplastic astrocytomas WHO grade III, 30 glioblastoma WHO grade IV, 28 oligodendrogliomas WHO grade II and 28 anaplastic oligodendrogliomas WHO grade III. Samples of normal brain were taken from two post mortems (female, 25 years of age: frontal, temporal and occipital cortex, thalamus, basal ganglia, pons, brain stem, cerebellum, pituitary, Dura; male 89 years of age: frontal and temporal cortex, Pons, cerebellum), a TMA containing tissue with neurodegenerative changes and one cortical biopsy with neurodegenerative alterations (female, 67 years of age).

For labelling, an automated stainer (Ventana Medical Systems) was used. The staining protocol comprised an antigen unmasking step (microwave treatment for 1 hour), incubation with the primary antibody for 30 minutes at 37°C (eIF-5A: rabbit monoclonal, Abcam ab32443, 1∶100; eIF-5A2: mouse monoclonal Abcam ab57421, 1∶50; DHS: rabbit polyclonal, Santa Cruz SC-67161, 1∶50; DOHH: custom made antibody (Eurogentec; briefly, rabbits were immunized with the following peptide: QDTSQEPMVRHEAGEC. Polyclonal antibodies were affinity purified against said peptide and provided in PBS with 0.01% thimerosal and 0.1% BSA) 1∶100, application of Ventana horseradish peroxidase detection kit, visualisation of bound antibodies with diaminobenzidine and counterstaining with alum-hematoxylin.

Determination of staining intensity was performed in 4 grades (0: none, 1: slight staining in up to 20% of cells, 2: moderate or strong staining in up to 50% of cells, 3: moderate to strong staining of >50% of cells) and only tumor cells were assessed. In normal brain tissue all tissue components were evaluated for antigen expression but staining intensities were not graded.

### RNA Extraction and qPCR

RNA from cells was extracted with peqGOLD Trifast (Peqlab) according to manufacturer’s instructions. After RNase treatment, one microgram of RNA per sample was transcribed into cDNA using M-MuLV reverse transcriptase (Fermentas) and 10 pmol poly-A primer (Invitrogen) for 60 minutes at 37°C and 10 minutes at 72°C.

The DyNAmo™ SYBR® Green qPCR Kit (Finnzymes) was used for quantitative PCR in 20 µl reactions according to the manufacturer’s instructions. Expression of human eIF-5A, -A2, DHS and DOHH was analyzed with the respective predesigned Quantitect primers (Qiagen, eIF-5A: QT00099911; eIF-5A2: QT00031283; DHS: QT00013888; DOHH: QT00235536) with RPLP0 (QT01839887) as housekeeping gene. One µl template cDNA was used per reaction. Cycling conditions for PCR were: 95°C for 7 minutes and 35 cycles of 95°C for 10 seconds and 60°C for 15 seconds. Relative expression was calculated using the 2^−ΔΔCT^ method [38].

### Western Blot

Cells were lysed with RIPA buffer complemented with protease inhibitor cocktail (Sigma-Aldrich) and 25 mM Na_3_VO_4_. After lysis for 10 minutes on ice, debris were pelleted by centrifugation at 21000×g and lysates were stored at −80°C. Western blotting was performed as described previously [28]. In brief, membranes were incubated with anti-eIF-5A antibody (Novus Biologicals) diluted 1∶5000 in 3% BSA, anti-DHS (Santa Cruz) 1∶500 in 1× RotiBlock (Roth) and anti-DOHH (Eurogentec) 1∶1000 in 5% skim milk (Roth) over night at 4°C. Anti-p53 (Santa Cruz), p21^Waf1/Cip1^, phospho-AKT (Ser473), AKT antibodies (Cell Signaling Technology) were diluted 1∶1000 in 5% BSA and applied to the membrane for one hour or overnight. Membranes were incubated with an anti-β-tubulin antibody (Oncogene, 1∶10000 in 3% BSA) for one hour as loading control. After washing, the membranes were incubated with anti-rabbit- (Cell Signaling Technology) or anti-mouse-HRP (Sigma-Aldrich) antibody (1∶10000 in PBST) for one hour. Aceglow chemiluminescence reagent (Peqlab) was used for visualization and images were collected with a CCD camera system (FUSION SL™, Peqlab). Membranes were stripped with Restore™ Western Blot Stripping Buffer (Thermo Scientific), washed and reprobed as described above. Hypusinated and non-hypusinated eIF-5A were detected by 2-dimensional-Western blots [39]. For this purpose, 25 µg of protein were diluted ad. 120 µl in urea lysis buffer (9 M urea, 4% CHAPS, 0.5% Resolyte [BDH Biochemicals, Poole, United Kingdom], 10 µg/ml bromophenol blue). First dimension IPG strips (7 cm, pH 7–14, GE Healthcare) were rehydrated with the protein solution overnight. Isoelectric focusing was carried out with the following parameters: 30 minutes at 500 V, 5000 Vh linear focussing at 5000 V followed by 5000 Vh “rapid” focussing at 5000 V and up to two hours storage at 500 V.

After focusing, IPG strips were equilibrated for 2×15 minutes in 6 M urea, 4% SDS, 50 mM Tris-HCl, pH 8.8, containing 1% DTT for the first or 4.8% iodoacetamide for the second period of equilibration. For the second dimension, samples were separated on a 12% SDS-PAGE and an anti-eIF-5A Western blot was performed as described above.

### Cell Proliferation Assay

G55T2 and U87-MG cell lines were seeded at 4×10^4^ cells/well in a 12-well plate. After 12 hours, GC7, TMZ or BCNU were added and cells were incubated for 48 or 72 hours. The maximum volume of vehicle (H_2_O for GC7, DMSO for TMZ and 50% EtOH for BCNU) served as vehicle control. Cells were harvested by trypsination with 300 µl trypsin/well followed by addition of 300 µl growth medium. The total volume was used for automated cell counting/viability analysis by trypan blue exclusion with a Vi-CELL Cell Viability Analyzers (Beckman Coulter).

### ^3^H-Spermidin Incorporation Assay

eIF-5A and eIF-5A2 are the only known proteins which incorporate the spermidine-derived aminobutyl moiety as hypusine. As described previously, this can be exploited to analyze and quantify hypusination by metabolic labelling of cells using ^3^H-labeled spermidine [40].

Briefly, 2,5×10^6^ U87-MG or G55T2 cells were seeded in a 10 cm cell culture dish. 25 µCi ^3^H-Spermdin (Hartmann Analytic) were added and cells were harvested after 48 hours. 200 µg of total protein were precipitated by adding one volume of 100% trichloric acid (TCA) to nine volumes of lysate and incubation for 5 minutes at −20°C and 15 minutes at 4°C. Proteins were spun down for five minutes at 16000×g. Protein pellets were washed twice with 1 ml of 10% TCA containing 1 mM spermidine and spermine followed by resuspension in 100 µl 1 N NaOH containing 1 mM spermidine and spermine respectively. Proteins were reprecipitated by adding 1 ml of 10% TCA, incubation for 30 minutes at 4°C and centrifugation. The activity in the supernatant was measured with a liquid scintillation analyzer (Tri-Carb 2900TR, PerkinElmer) and the washing steps were repeated until no radioactivity >200 dps was detectable. After resuspension of proteins in 100 µl 1 N NaOH, radioactivity of total proteins was measured as described above.

**Figure 1:**
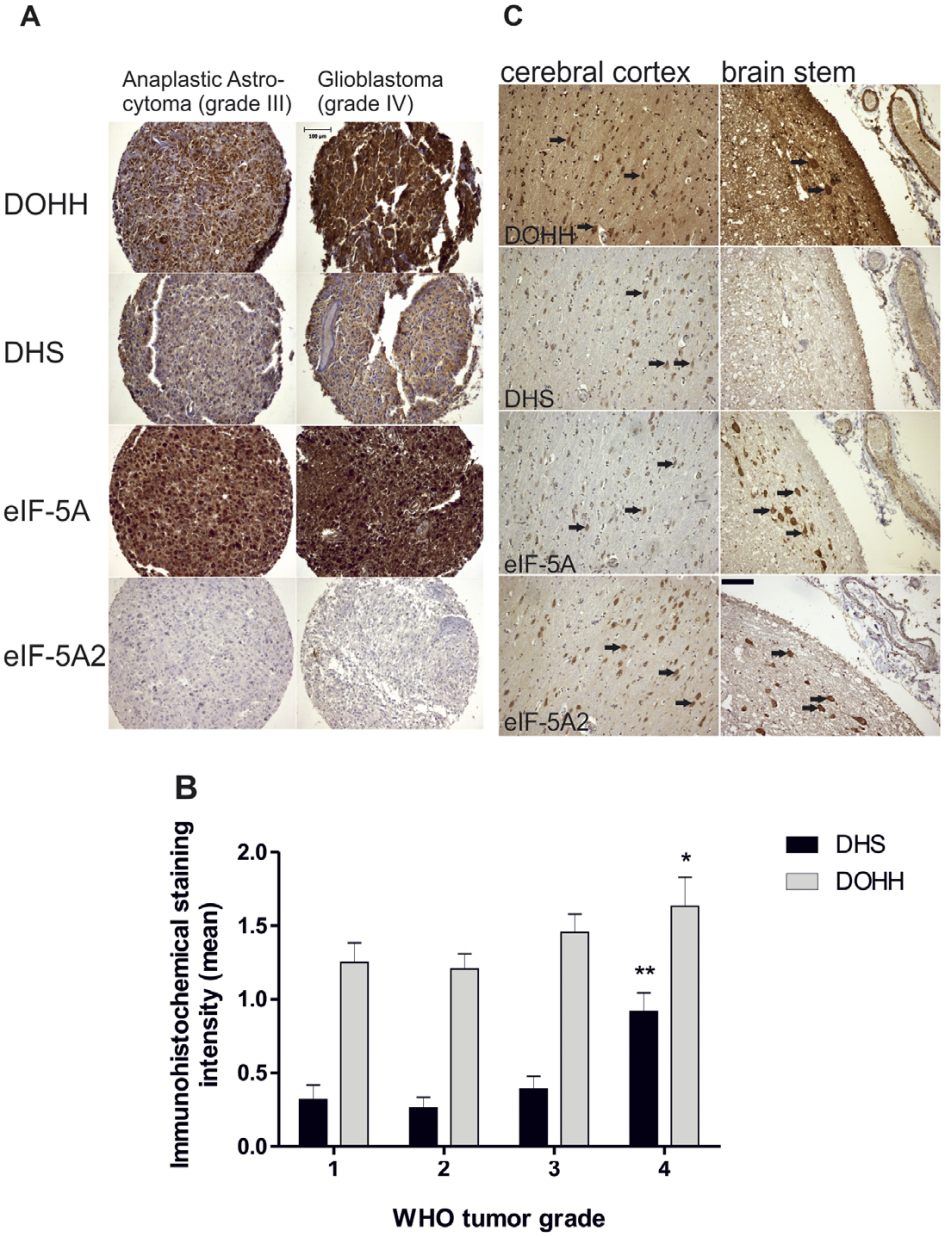
eIF-5A, DHS and DOHH are expressed in gliomas of different grades. (A and B) DOHH and DHS expression significantly correlate with tumor grade (mean staining intensity ± SEM; *:p<0.01 and **:p<0.001, respectively; two-sided non-parametric Kendall tau-b correlations). (C) In the brain mainly large neurons (arrows) such as in the pyramidal layer of the cortex (left column) or in the brain stem (right column, arrows point to neurons of the nuclei arcuati) were labelled. The scale bar represents 100 µm.

### Lentiviral Transduction

Small hairpin RNAs against human eIF-5A mRNA in a lentiviral vector (pLKO.1-puro) were purchased from Sigma-Aldrich (MISSION shRNA clones TRCN0000062551, TRCN0000062552, TRCN0000062548, TRCN0000062549, TRCN0000062550. Similar vectors expressing shRNAs against DHS mRNA (Sigma-Aldrich) were a kind gift by Dr. Jan Chemnitz (Heinrich Pette Institute, Hamburg). The packaging plasmids pMDLg/pRRE (Gag/Pol), pRSV-Rev (Rev) and phCMV-VSV-G (envelope) were used for production of lentiviral particles in 293T packaging cells as described elsewhere [41]. Supernatants containing viral particles were passed trough a 0.45 µM filter and applied to the target cells for 24 hours supplemented with 8 µg/ml polybrene. For selection of transduced G55T2 and U87-MG cells, cultures were grown in presence of 2.5 µg/ml puromycin for two days.

Lentiviral gene ontology (LeGO) vectors [42] have been used to express human wild type p53 (Accession number NM_000546) in G55T2 cells [43]. Besides p53, the bicistronic vector expressed a combined drug selectable fluorescent marker [44], consisting of Cerulean fluorescent protein (cyan) and Puromycin N-acetyl-transferase, conferring resistance to puromycin. The corresponding control vector expressed the combined marker only. Maps of both vectors are provided (see Figure S1).

**Figure 2:**
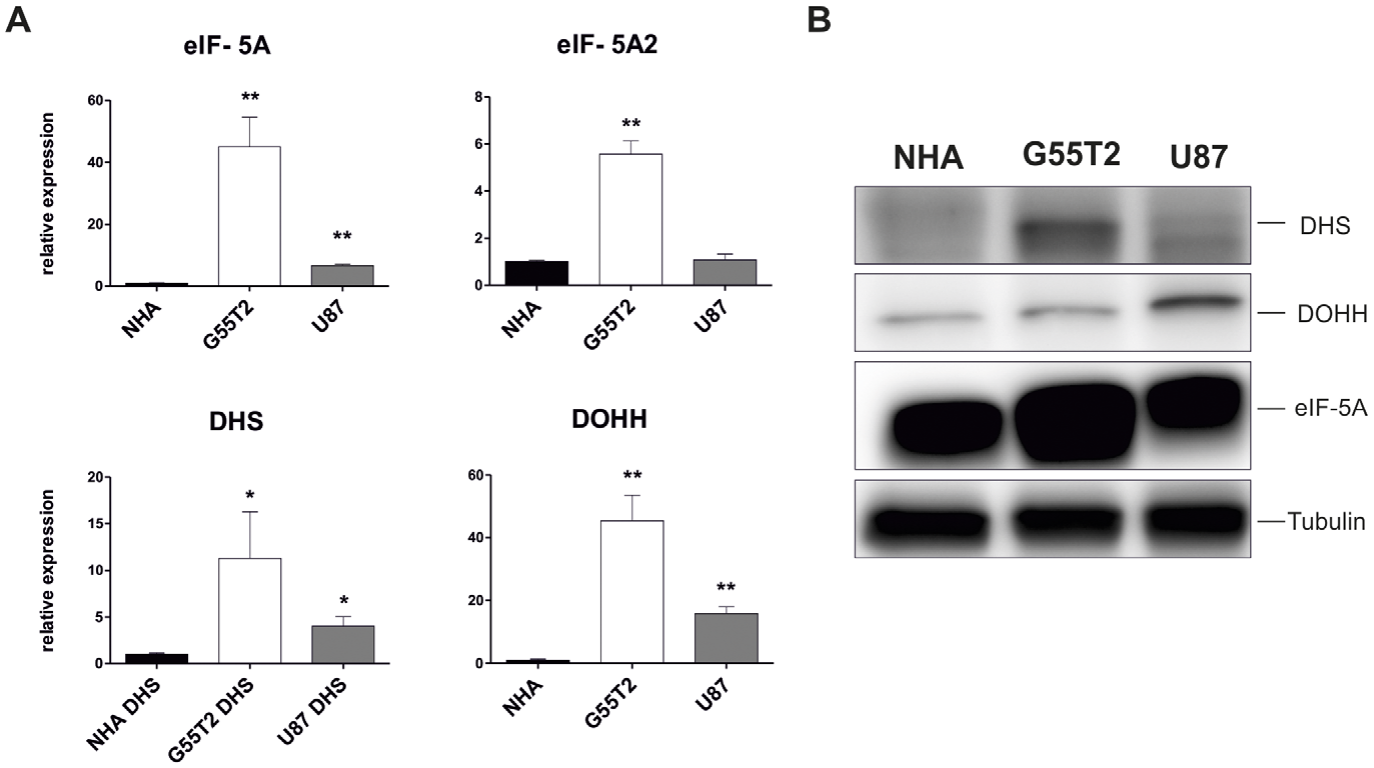
eIF-5A, DHS and DOHH are overexpressed in the glioblastoma cell lines G55T2 and U87-MG. (A) Expression levels of eIF-5A, -A2, DHS and DOHH were analysed by qPCR (mean ±SD, n = 3; *:P<0.05; **:P<0.001 ) in primary normal human astrocytes (NHA) compared to G55T2 and U87-MG cell lines. SYBR green Ct values were normalized against GAPDH expression and calculated using the 2^−ΔΔCT^ method. (B) Overexpression of eIF-5A, DHS and DOHH was confirmed by immunoblotting with whole cell lysates (35 µg protein per sample).

### Determination of Necrotic and Apoptotic Cells, Cell Cycle Analysis and Detection of Senescence-associated β-galactosidase Positive Cells

For measuring necrosis/apoptosis and cell cycle, 5×10^5^ cells were seeded in 6 cm dishes and 50 µM GC7, 150 µM TMZ or 20 µM BCNU alone or TMZ/BCNU in combination with GC7 were added after 12 hours of preincubation. Cells were harvested after 48 hours of incubation, and cell cycle or the sub-G_1_ population was analyzed by propidium iodide (PI) staining followed by flow cytometry (FACSCalibur, Becton-Dickinson) as described by Riccardi and Nicoletti [45]. Cell cycle analysis was performed using the FlowJo software (Tree Star). The cleavage of caspase-3 as a marker for apoptosis was determined by FACS using a cleaved caspase-3 specific, PE-linked antibody (BD Bioscience), after cells were incubated with GC7 as described above and fixed with 2% paraformaldehyde and 90% methanol. For detection of apoptotic cells by TUNEL assays, 1×10^4^ U87-MG or G55T2 cells per well were seeded on a 4-well chamber slide and incubated with 50 µM, 100 µM GC7 or vehicle for 72 hours. The assay was performed with the In Situ Cell Death Detection Kit, POD (Roche) according to the manufacturers’ instructions. Senescence-associated activity of lysosomal β-galactosidase was investigated as described in [46]. Microphotographs of cells were taken with an Axiovert 25 microscope and an AxioCam MRc camera (Zeiss) at 100× or 320× magnification.

**Figure 3:**
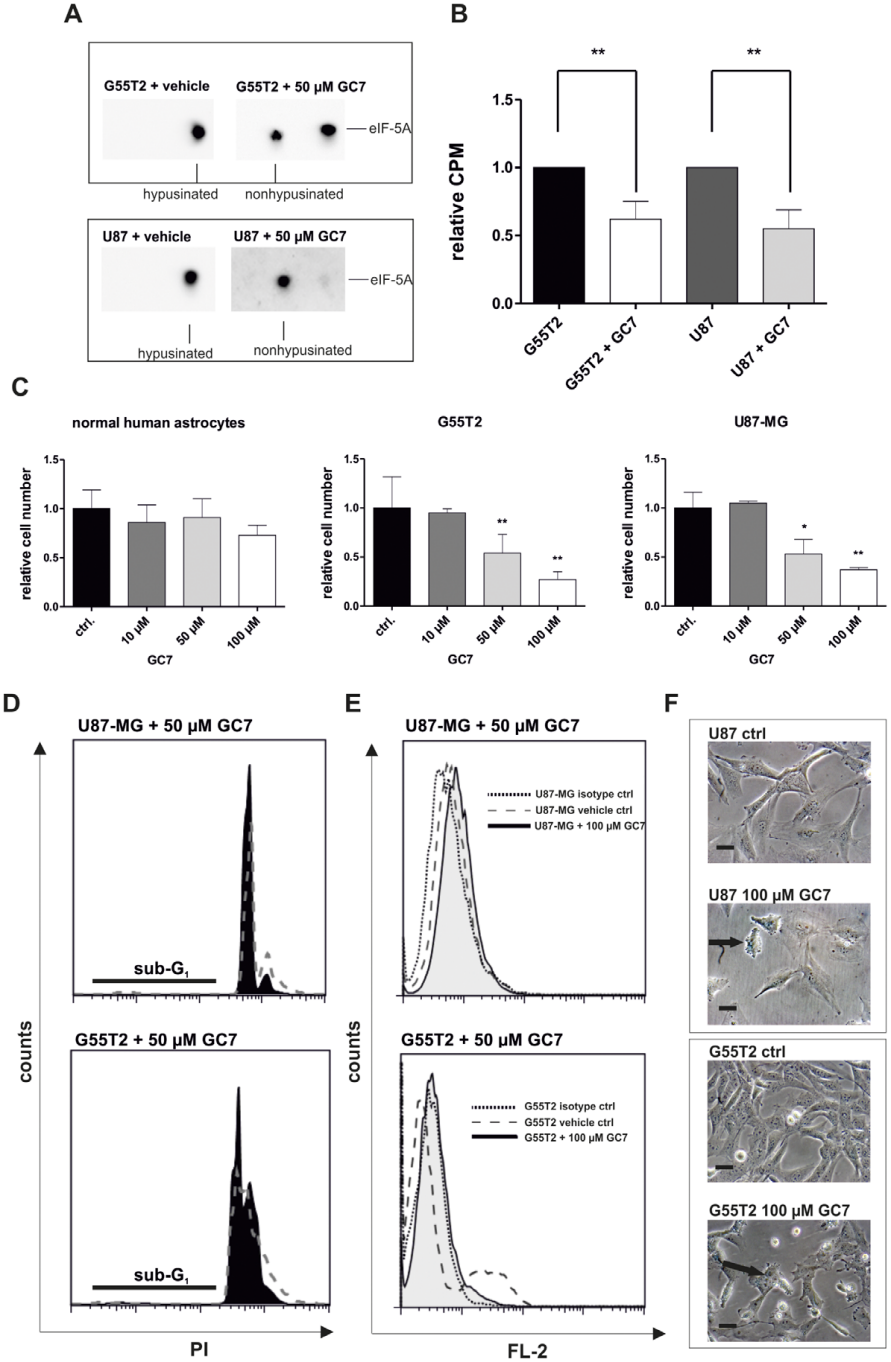
Effect of GC7 on proliferation, hypusine status and viability in G55T2 and U87-MG cells. (A) Cells were incubated with 50 µM GC7 or vehicle for 48 hours. The effect of GC7 on eIF-5A hypusination was determined by a change of the pI of eIF-5A, visualized by 2D-Western blot using an antibody against eIF-5A (25 µg total protein, pH 4–7 first dimension strips). (B) To verify reduced hypusination, cells were co-incubated with GC7 and 25 µCi ^3^H-spermidine. 200 µg of total cellular proteins were precipitated with 10% TCA and excessive radioactivity was removed by washing. The protein pellet was resuspended in 1 N NaOH and the activity of ^3^H, incorporated into hypusine, was measured. (C) Normal human astrocytes (NHA), G55T2 and U87-MG cells were treated with the indicated concentrations of GC7 for 72 hours. Viable cells were counted by trypan blue exclusion and data are expressed as relative cell numbers compared to mock treated cells (mean ±SD, *n = *6). Significantly different cell numbers compared to controls are indicated by asterisks (*:P<0.05; **:P<0.001). (D) U87-MG and G55T2 cells were treated with 50 µM GC7 (black area) or water (dotted area) for 72 hours, fixed in 70% ethanol and stained with PI. Apoptotic/necrotic (sub-G_1_) populations were determined by FACS analysis after PI staining. (E) U87-MG and G55T2 cells were treated with 50 µM GC7 (gray area) or water (dashed area) and stained with antibodies against cleaved caspase-3 or isotype control (dotted area) to determine the rate of ongoing apoptosis upon treatment. (F) Microphotograph of mock and GC7 treated U87-MG and G55T2 cells after five days of drug treatment. The photos were taken at x100 magnification. Arrows mark examples of morphologic changes upon treatment. Each bar represents 10 µm.

### Detection of Apoptosis by Annexin-V/Propidium Iodide Staining

U87-MG and G55T2 cells were seeded in 10 cm cell culture dishes (0.5×10^6^ per dish) and treated with 150 µM TMZ, 50 µM GC7 or a combination of both as indicated above. Cells were then harvested and stained with Annexin-V-FITC and PI (BD Bioscience) according to the manufacturer’s instructions. Quantitative analysis of viable, apoptotic and dead cells was performed by flow cytometry, with 20.000 events acquired from each sample.

### Statistics

Statistical analysis for immunohistochemistry was performed using the SPSS software version 15. Patient age, gender, tumor type (astrocytoma vs. oligodendroglioma), tumor grade and immunohistochemical staining intensity were correlated. Comparisons involving metric variables were analysed according to Pearson whereas all ordinal scaled variables were compared using the Kendall tau-b test. For other experiments we performed unpaired two-tailed t-tests, using GraphPad Prism 5.01 (GraphPad Software).

**Figure 4:**
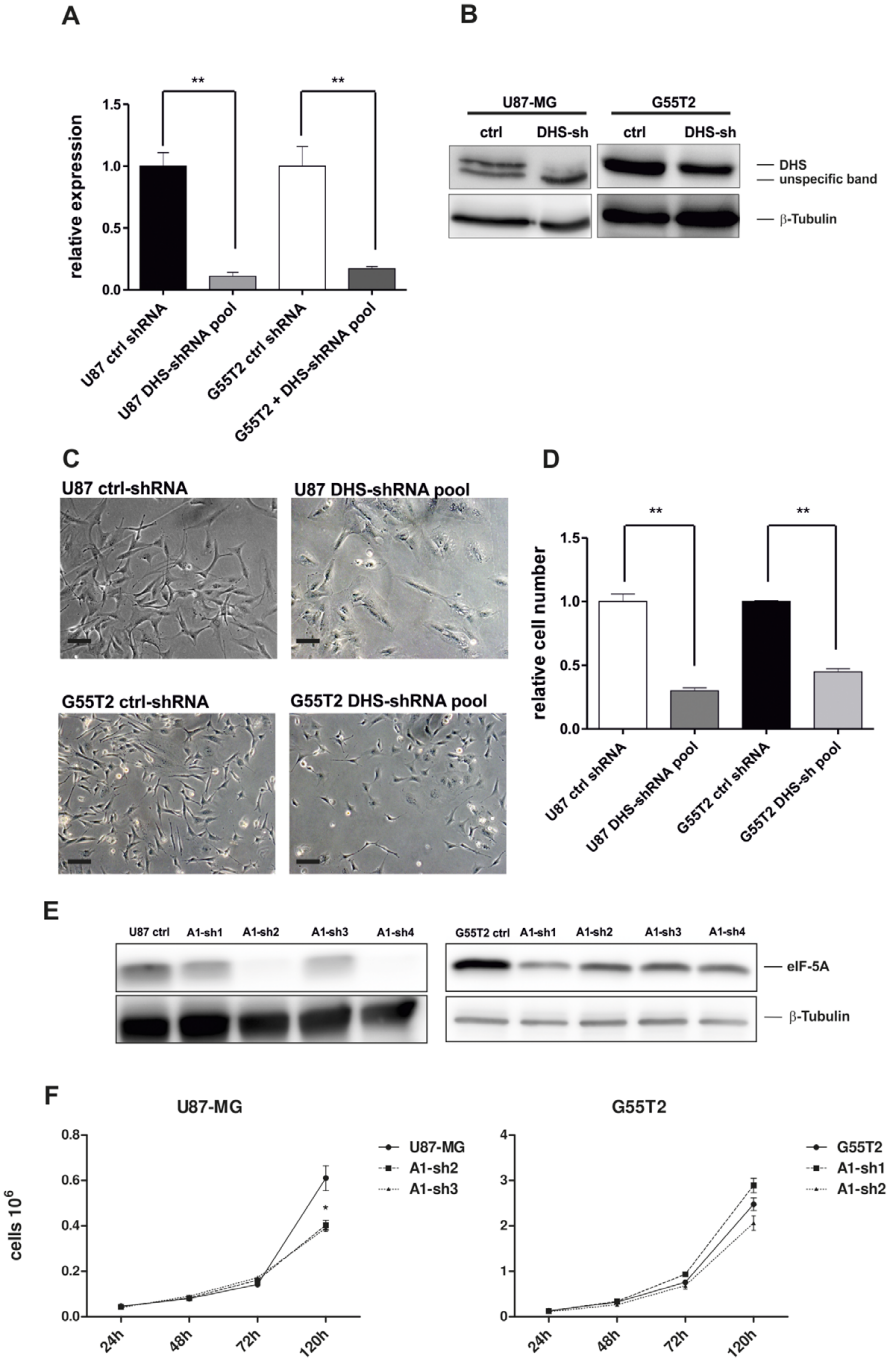
Knockdown of DHS mRNA in U87-MG and G55T2 cell lines by gene specific shRNAs. (A) Relative DHS-mRNA expression in G55T2 and U87-MG cells after lentiviral transduction with a scrambled shRNA (G55T2-ctrl and U87 ctrl) or DHS-specific shRNA pools normalized against GAPDH (mean±SD, *n = *3). (B) Immunoblot (30 µg total protein per sample) against DHS and β-Tubulin in G55T2 and U87-MG cells after lentiviral transduction with a scrambled shRNA (G55T2-ctrl and U87-ctrl) or DHS-specific shRNAs (G55T2 DHS-sh and U87 DHS-sh) after two days of puromycin selection. (C) Glioblastoma cell lines after transduction with a scrambled control shRNA or an shRNA-pool against DHS (100× magnification) with 100 µm scale bars. (D) Proliferation of transduced cells measured by trypan blue exclusion over five days. Significantly different cell numbers or DHS expression compared to controls are indicated (*:P<0.05; **:P<0.001). (E) Immunoblot results of U87-MG and G55T2 cells after transduction with a scrambled control shRNA (ctrl) or four different shRNAs against eIF-5A (30 µg total protein). Transduced cells were selected by incubation with puromycin for two days, cell lysates were obtained seven days post-selection. (F) Proliferative capacity of transduced cells with strong (A1-sh2 for U87-MG and A1-sh1 for G55T2) or moderate (A1-sh3 for U87-MG and A1-sh2 for G55T2) eIF-5A knockdown was determined by trypan blue exclusion cell counting over five days. Significantly different cell numbers compared to controls are marked by an asterisk (*:p<0.05).

### Ethics Statement

The immunohistochemical study was performed on archival tissue that was processed and diagnosed in the Institute of Neuropathology, University Medical Center Hamburg-Eppendorf, Germany. According to the law for hospitals in Hamburg (Hamburger Krankenhausgesetz), Germany, samples of closed cases that were processed and investigated in a medical institution may be used anonymously for research purposes by the same institution without need for a separate written informed consent from the patients.

**Figure 5:**
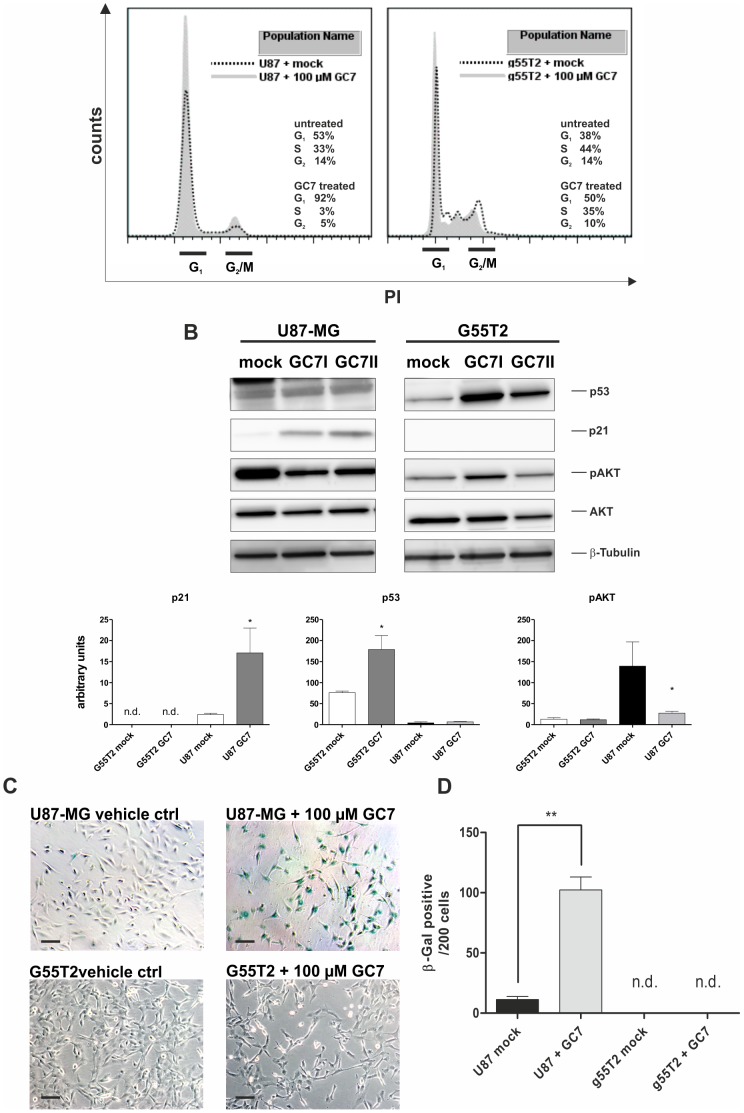
Induction of cell cycle arrest and cellular senescence by GC7. GC7-treatment of U87-MG and G55T2 cells leads to a higher proportion of cells in G_1_-cell cycle phase, upregulation of p21*^Waf1/Cip1^*, downregulation of phospho-AKT and activity of senescence associate β-galactosidase. (A) After incubation cells with 100 µM GC7, the cell cycle profile was compare to untreated cells by PI staining and FACS analysis. (B and C) Total protein lysates (35 µg) of G55T2 and U87-MG cells after treatment with 100 µM GC7 (duplicates) or water were immunoblotted for p53, p21*^Waf1/Cip1^*, phospho-AKT, AKT and β-tubulin. Signals were quantified after normalization against β-tubulin. (D and E) Microphotograph at x100 magnification of mock and GC7 treated U87-MG cells after SA-β-galactosidase assay. Scale bars represents 100 µm. Quantification of SA-β-gal^+^ cells (triplicates, 200 cells per sample were counted; n.d. = no SA-β-gal^+^ cells detected).

## Results

### eIF-5A, DHS and DOHH are Overexpressed in Glioma Tissue Samples with Different Grades and in Glioblastoma Cell Lines

Using immunohistochemistry (IHC), we found elevated expression levels of DHS, DOHH and eIF-5A in 173 glioma samples with different grades (Table S1). eIF-5A was demonstrated to be overexpressed in all tumors (Figure 1A). This observation was independent from tumor grading. The isoform eIF-5A2 was only detected in one single tumor. Interestingly, DHS and DOHH were significantly upregulated in glioblastoma samples compared to tumors of grade I–III (Figure 1A, Table S2). To estimate the expression of these proteins in normal brain, we performed IHC on tissue from two healthy donors. As depicted in Figure 1C, the anti-DOHH antibody immunolabelled neurons, oligodendroglia, astrocytes, fibroblasts, endothelia, smooth muscle cells of vessels and pituitary tissue whereas the other three antibodies did not bind to normal astrocytes. In detail, DHS expression was detected in neurons, ependyma, fibroblasts and pituitary. eIF-5A was also found in neurons, ependyma and pituitary and additionally in meningothelial cells, choroid plexus and some reactive astrocytes. Notably, we could not detected eIF-5A expression in the majority of the glia cells. The second isoform eIF-5A2 was present in neurons, ependyma, meningothelial cells, fibroblasts, smooth muscle cells and pituitary (Figure 1C). In order to establish an *in vitro* model for further functional characterisation of the hypusine modification in gliomas, we analysed the expression of eIF-5A, eIF-5A2, DHS and DOHH in different cell lines. Determination of mRNA and protein levels of eIF-5A, DHS and DOHH in G55T2 and U87-MG cell lines showed overexpression of eIF-5A and the two hypusine forming enzymes compared to primary human astrocytes (Figure 2A. Overexpression of the eIF-5A2 isoform was detectable in G55T2, but not in U87-MG cells. The expression level of all four analysed mRNAs was highest in G55T2 cells, whereas in U87-MG cells it was not as pronounced but statistically significant. These findings were confirmed on protein level, however contrary to qPCR results, DOHH protein levels seemed to be higher in U87-MG cells than in G55T2 (Figure 2B).

**Figure 6:**
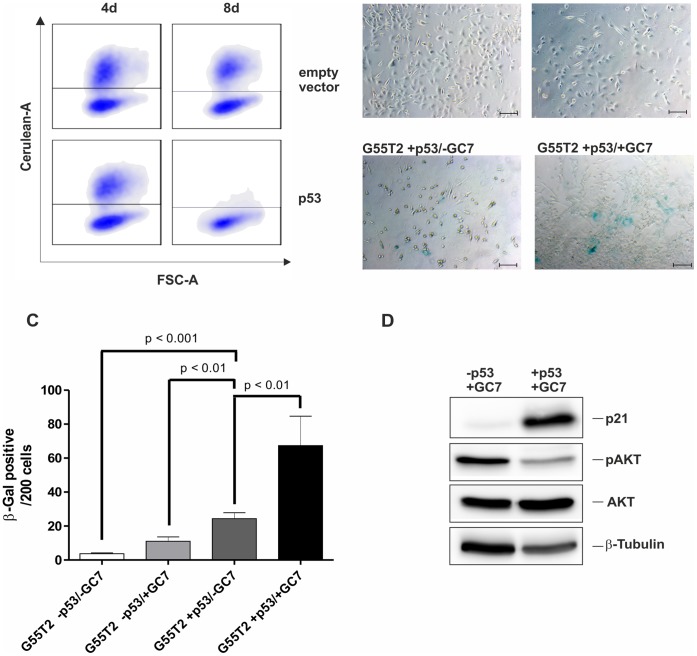
Restauration of p53 in G55T2 cells leads to increased premature senescence by DHS inhibition. (A) Empty vector- and p53-transduced G55T2 populations were monitored by FACS, 4 and 8 days post transduction. (B and C) GC7 and mock treated G55T2 cells +/− p53^wt^ were stained for SA-β-galactosidase activity. Microphotographs were taken at x100 magnification. Scale bars represents 100 µm. SA-β-gal^+^ cells were determined in triplicates, 200 cells per sample were counted. (D) Expression of p21*^Waf1/Cip1^*, phospho-AKT, AKT and β-tubulin in GC7 treated G55T2 cells +/− p53^wt^ was analysed by immunoblotting.

### Inhibition of DHS by GC7 Induces Antiproliferative Effects *in vitro*

Based on this significant overexpression of the hypusine associated proteins in gliomas and glioma cell lines, we asked whether hypusination inhibitors (HI) were able to inhibit the proliferation of glioma cell lines in vitro. In a first set of experiments we confirmed that the known DHS inhibitor GC7 inhibited the hypusine synthesis *in vitro* [47]. For this purpose we used 2D-Western blotting of eIF-5A and ^3^H-Spermidin incorporation assays. Hypusin-containing eIF-5A can be distinguished by 2D-Western blot, since the non-hypusinated form is characterized by a different (more acidic) isoelectric point compared to its hypusinated counterpart. In the cell lines G55T2 and U87-MG, the majority of eIF-5A is hypusinated, indicated by a single protein spot (Figure 3A). The posttranslational hypusine modification of eIF-5A can be prevented by treating cells with the spermidine analogue GC7, which inhibits DHS by competitive replacement of its native spermidine substrate. Treatment of glioblastoma cells with GC7 *in vitro* resulted in a reduced amount of modified eIF-5A (50% in G55T2 and 45% in U87-MG) indicated by a second, more acidic eIF-5A spot in 2D-Western blot (Figure 3A). This was verified by a lower rate of ^3^H-spermidine incorporation in G55T2 and U87-MG cells (Figure 3B). The inhibition of DHS with increasing doses of GC7 showed a concentration-dependent reduction of proliferation in glioblastoma cells (Figure 3C). The effect of GC7 was already detectable after 48 h hours (data not shown) with a ∼50% reduction of cell proliferation at 50 µM after 72 hours compared to untreated cells. Noteworthy, normal human astrocytes showed no significantly reduced proliferation within 72 hours with the lowest growth at 100 µM (73% compared to untreated cells). We could not detect an increase of apoptotic or necrotic cells by trypan exclusion (data not shown), no effect on the sub-G_1_ fraction of PI stained cells and no increase of caspase-3 positive cells (Figure 3D and E) or TUNEL positive cells (data not shown) when cells were treated with GC7. GC7 treated GBM cells showed morphological changes after two days (Figure 3F). Interestingly, U87-MG cells became flattened or round and detached. In contrast, G55T2 cells did not become flattened. Instead they started to accumulate vesicles in the cytoplasm.

**Figure 7:**
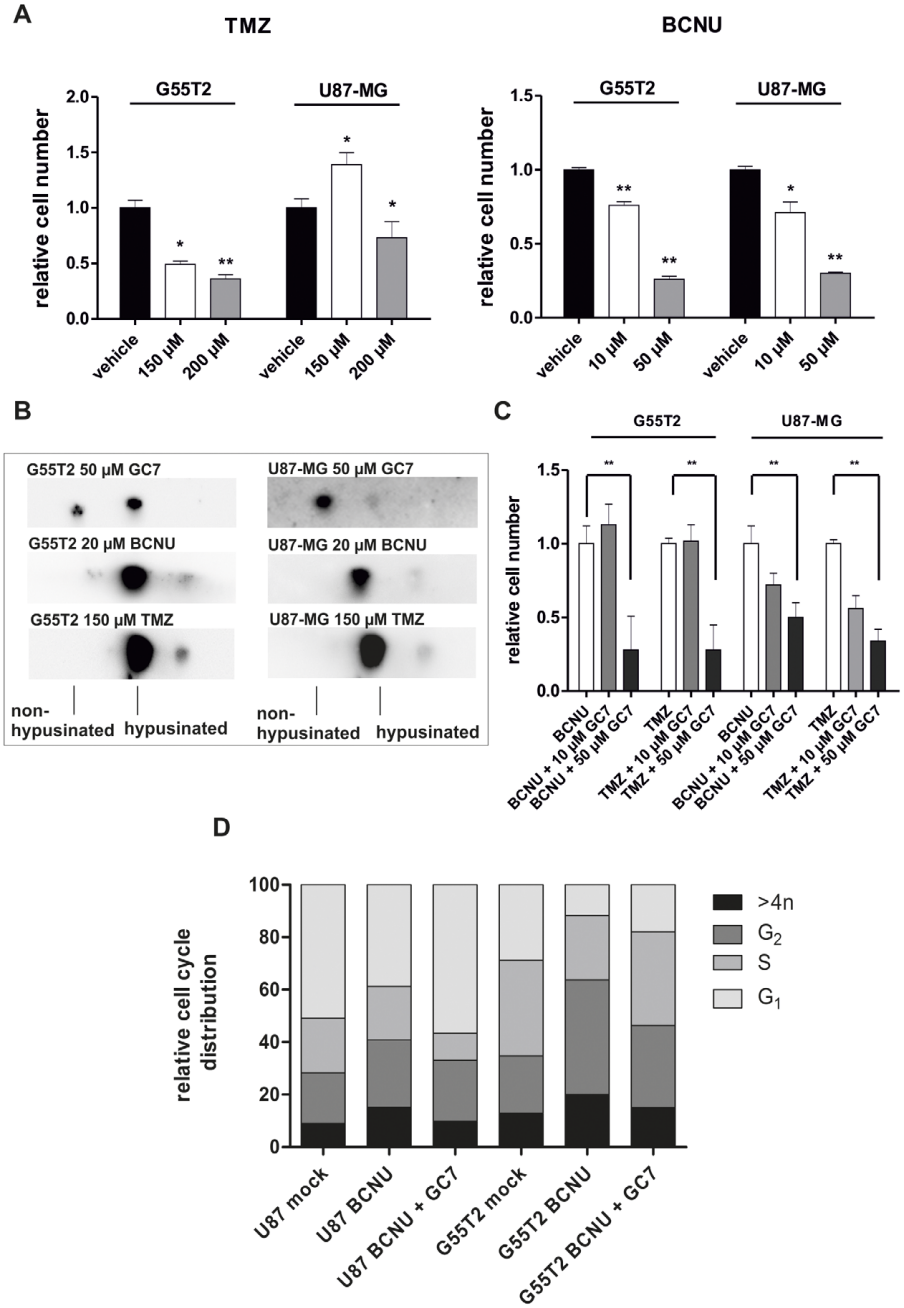
Co-treatment of glioblastoma cells with alkylating agents and GC7 has an additive antiproliferative effect. (A) G55T2 or U87-MG cells were treated with TMZ (150 µM and 200 µM) or BCNU (10 µM and 50 µM) for 72 hours. Cells were analyzed by trypan exlusion and automated cell counting. (B) The direct effect of alkylating drugs on the synthesis of hypusinated eIF-5A was investigated by treating cells with TMZ or BCNU and comparing the modification state of eIF-5A to GC7 treated cells by 2D-Western blot against eIF-5A. (C) Cells were treated with BCNU/TMZ alone or together with two GC7 concentrations. Effects on the proliferative capacity of treated glioblastoma cells were measured by trypan blue exclusion after 72 hours. Significantly different cell numbers compared to controls are indicated by asterisks (*:P<0.05; **:P<0.001). (D) The influence of vehicle, TMZ/BCNU single and TMZ/BCNU and GC7 co-treatment on the cell cycle of U87-MG cells was evaluated by PI staining and subsequent FACS analysis.

### Knock-down of eIF-5A and DHS Impairs Proliferation of Glioma Cells *in vitro*

Since GC7 is a polyamine analogue, it may cause physiological responses which cannot be attributed to the inhibited hypusine formation. To exclude such drug-induced side-effects, DHS and eIF-5A were knocked down by stable lentiviral expression of shRNAs in G55T2 and U87-MG cells. A pool of four DHS shRNA vectors could significantly reduce DHS expression on mRNA and protein level (Figure 4A and B) and resulted in morphological changes comparable to glioblastoma cells treated with higher doses (50–100 µM) of GC7, although the effect in G55T2 cells was less pronounced when directly compared to U87-MG (Figure 4C). As expected, the proliferation was significantly reduced to 30% for U87-MG (p<0.001) and 45% for G55T2 (p<0.001) compared to cells transduced with a scrambled shRNA (Figure 4D). Knockdown of eIF-5A with four different shRNAs resulted in a strong to moderate (U87-MG) or moderate to low (G55T2) knockdown compared to cells transduced with a scrambled shRNA (Figure 4E). Both U87-MG lines (with strong or moderate knockdown) show a significantly lower proliferation after five days, whereas the achieved eIF-5A knockdown in G55T2 cells was not sufficient to induce an antiproliferative effect (Figure 4F).

**Figure 8:**
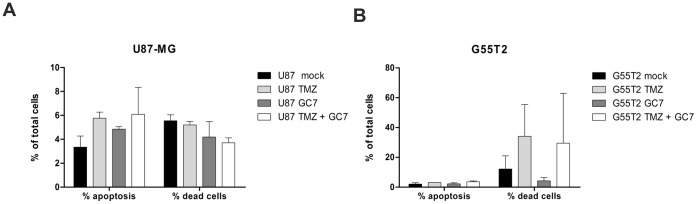
Effects of a combination of GC7 with TMZ on induction of apoptosis. (A) G55T2 or (B) U87-MG cells were treated with TMZ (150 µM) and/or GC7 (50 µM) for 72 hours.No significant cooperative effects of both drug in induction of apoptosis have been detected. Fractions of viable, early apoptotic and dead cells were assayed by an Annexin-V/PI double staining and FACS analysis (no significant differences; p>0.05 for all experiments).

### GC7 Treatment Induces Premature Senescence in U87-MG Cells

Inhibition of hypusine formation has been reported to have an effect on the cell cycle progression in some cell types, e.g. yeast cells, murine neuroblastoma and erythroleukemia cell lines [48]. We could observe an increase of U87-MG cells in the G_1_-phase from 53% to 92% and a reduction of cells in S- and G_2_-phase (from 33% to 3% and 14% to 5% respectively) after treatment with 100 µM GC7 (Figure 5A). This increase of cells in the G_1_-phase and their morphology led us to the assumption that cells enter a terminal cell cycle arrest, reminiscent of premature senescence. Indeed, in a senescence-associated (SA) β-galactosidase staining, GC7-treated U87-MG cells were positive. We observed a 27-fold increase of SA-β-gal^+^ in GC7 treated cells compared to mock-treated cells (Figure 5C and D). This phenotype was supported by the observation that p21*^Waf1/Cip1^*, one of the key regulators of the cell cycle, was strongly upregulated 15-fold (Figure 5B). This was accompanied by a 4-fold reduction of phosphorylated AKT (an inhibitor of the p21*^Waf1/Cip1^* mediated antiproliferative effect). In contrast, no senescent phenotype could be observed in G55T2 cells. Those cells were negative for the SA-β-galactosidase staining and the G_1_-population increased only from 38% to only 50%, whereas the S- and G_2_-populations dropped from 44% and 14% to 35% and 10% respectively. In contrast to U87-MG cells, p53 was upregulated, but p21*^Waf1/Cip^* was not detectable with or without GC7 in G55T2 cells and no change in phosphorylated AKT occurred.

### Restoration of p53^wt^ Enables Premature Senescence in G55T2 Cells after DHS Inhibition

In order to further investigate the role of p53 in hypusine-dependent induction of senescence we expressed wild type p53 in G55T2 cells. Transduction of G55T2 cells with p53 lead to cell death of the transduced population (data not shown). This p53^wt+^ population was reduced 10-fold within eight days post transduction, whereas cells transduced with an empty control vector remained viable (Figure 6A), indicating p53-sensitivity of the p53-mutated G55T2 cells. Treatment with 100 µM GC7 for 72 hours, starting 24 hours post transduction, lead to an increase of cells in premature senescence as assayed by SA-β-galactosidase staining (Figure 6B and C; 4% untreated p53^−^ vs. 30% p53^wt+^/+GC7). However, even in the absence of GC7, a significant fraction of the p53-transduced cells was SA-β-galactosidase^+^ compared to p53^-^ cells (with or without GC7). Nevertheless, treatment with GC7 increased the amount of SA-β-galactosidase^+^ cells significantly (8% vs. 30%). Additionally, the application of GC7 for 72 hours increased the expression of p21 in p53^wt+^ cells, whereas in cells transduced with the empty vector, p21 expression remained low (Figure 6D). As observed in U87-MG cells, induction of senescence and p21 up-regulation was accompanied by a reduction of phosphorylated AKT.

### Co-treatment of Glioblastoma Cells with TMZ/BCNU and GC7 has an Additive Antiproliferative Effect

As expected, U87-MG and G55T2 cells were both susceptible to treatment with TMZ and BCNU and have shown a dose dependent reduction of proliferation, with the exception of U87-MG cells at 150 µM TMZ (Figure 7A). Both cell lines reacted similar to BCNU with cell numbers <50% compared to controls after 72 hours treated with 50 µM BCNU. Clearly, the cell line G55T2 was more sensitive to TMZ (Figure 7A). 72 hours after administration of 150 µM TMZ, cell numbers were 50% less as compared to controls, whereas in U87-MG cells even 200 µM could reduce proliferation to only about 70% (Figure 7A). To rule out a potential effect of alkylating drugs on the hypusine pathway, we analysed the hypusine status of eIF-5A in cells treated with 150 µM TMZ or 20 µM BCNU compared to cells treated with 50 µM GC7. As described before, inhibition of DHS results in accumulation of unhypusinated eIF-5A. As shown in Figure 7B, TMZ or BCNU had no comparable effect and no accumulation of non-modified eIF-5A was detectable.

Co-treatment of G55T2 cells with 150 µM TMZ and 10 µM GC7 did not result in an enhanced antiproliferative effect, but co-treatment with 50 µM GC7 resulted in a significant (p<0.001) reduction of proliferation to 28% as compared to cells treated with TMZ alone (Figure 7C). Co-treatment with 20 µM BCNU and 50 µM GC7 yielded a similar result with a reduction of cell number to 44% as compared to cells incubated with 20 µM BNCU alone (p<0.001). The reaction of U87-MG cells was slightly different, with a cell number of 70% (concurrent treatment with 20 µM BCNU and 10 µM GC7; p<0.05) and 50% (20 µM BCNU and 50 µM GC7; p<0.001) as compared to cells incubated with solely BCNU. Co-treatment with TMZ and GC7 yielded a lower proliferation rate comparable to G55T2 cells (56% with 10 µM GC7 and 34% with 50 µM GC7 respectively). In this study, treatment of GBM cells with alkylating agents for 72 hours resulted in cell cycle arrest in G_2_-M phase (TMZ data not shown) with a minor increase of the sub-G_1_ population in case of BCNU treatment (data not shown) and an accumulation of hyperploid (>4n) cells, mimicking the results described previously [49]–[52] (Figure 7D). Since inhibition of hypusine synthesis lead to rise of the G_1_-fraction and TMZ/BCNU treatment to G_2_-M arrest, a combination of those agents caused a mixed phenotype within 72 hours of incubation. The fraction of cells with haploid DNA content in double treated cells was smaller or equal compared to control cells, but higher compared to BCNU treated ones, reflecting the increase in the G_1_-population caused by GC7. The G_2_-M population was equal in cells incubated with BCNU alone or together with GC7. However, in BCNU/GC7 double treated cells, the poly-n population was reduced considerably compared to single treated cells (Figure 7D). Although GC7 alone was not able to induce apoptosis in our cellular model, we wanted to explore a possible proapototic effect in TMZ-stressed cells. Therefore, we analysed the accumulation of early apoptotic and late apoptotic/dead cells by Annexin-V/PI double staining in GBM cells after single treatment with TMZ, GC7 or a combination of both compounds (Figure 8A–B). As depicted in Figure 8 treatment with TMZ and GC7 alone or in combination did not result in a significant induction of apoptosis neither in U87-MG (Figure 8A) nor in G55T2 cells (Figure 8B).

## Discussion

The pro-proliferative properties of eIF-5A and antiproliferative effects of inhibited hypusine formation have been firmly established in the past (e.g. by Park et al. [53]). To evaluate a possible role of eIF-5A and the hypusine synthesizing enzymes in glioma, we analysed samples from 173 glioma patients. We found a general eIF-5A overexpression in tumor cells and an upregulation of DHS and DOHH in glioblastomas compared to tumors of grade I–III. These findings may indicate a role for hypusinated eIF-5A in the formation or progression of gliomas, especially in glioblastomas. This hypothesis is substantiated by the finding that in samples of two non-glioma patients, eIF-5A, eIF-5A2 and DHS expression was mainly detectable in persisting neurons, ependymal and some other brain tissues, but rarely or not at all in astrocytes. This underscores the role of hypusinated eIF-5A in transformation or propagation of tumor cells, since glioblastomas are possibly derived from progenitors along the neural stem cell-astrocytic axis or de-differentiated astrocytes [54]. Further, more recent gene expression studies have revealed that glioma-patients with a high expression of eIF-5A have a lower probability of survival, compared to patients with an intermediate expression (National Cancer Institute REMBRANDT database www.caintegrator.nci.nih.gov/rembrandt; *p = *0.043; mean of all reporters; Figure S2). Additionally, in a dataset available through “The Cancer Genome Atlas Research Network”, two transcripts of eIF-5A are overexpressed in GBM (*p = *0.0344 and *p = *0.000181) [5]. The main transcript of DHS is upregulated as well (*p = *0.0408). In contrast, the expression of DOHH is not altered. However, two miRNAs putatively targeting the DOHH mRNA are strongly downregulated in tumors (both *p*<0.0001). That indicates the biological significance of the hypusine modification systems in glioblastoma.

To evaluate the possible role of functional eIF-5A as a potential therapeutic target in glioblastomas, we treated tumor derived cell lines with GC7, a well established specific inhibitor of desoxyhypusine synthase (DHS), and compared its antiproliferative effect to treated primary astrocytes. Clearly, GC7 reduced the proliferation of glioblastoma cells significantly and caused changes in cell morphology. The effect of GC7 on the two glioblastoma cell lines analysed in this study appears to be rather cytostatic than cytotoxic. Strikingly, in U87-MG cells we could identify premature senescence as a potential mechanism of observed reduction in cell growth. U87-MG cells are positive for senescence-associated β-galactosidase and upregulation of the p53-downstream effector p21*^Waf1/Cip1^* (a cyclin dependent kinase inhibitor and cell cycle regulator) underlines the manifest G_1_-cell cycle arrest in those cells. The nature of a senescent phenotype is complex and depends on the cellular context as well as the senescence-inducing stimulus [55]. p21*^Waf1/Cip1^* expression can be regulated in a p53 dependent manner and it is a key regulator of senescence [56]. Since the U87-MG cells have deleted p16 and p14^ARF^ [57], which are also important regulators of senescence, we can assume that the observed senescence phenotype relies on p21 as major regulator. This finding is reinforced by the observed reduction of pAKT in GC7-treated U87-MG cells. Phosphorylated AKT has been described as an inhibitor of p21*^Waf1/Cip1^* and its reduced activity is likely to contribute to the p21*^Waf1/Cip1^* mediated senescence [58]. In contrast to above findings, G55T2 cells, susceptible to GC7 as well, showed no signs of senescence. Since these cells exhibit mutated p53, the downstream signal transduction does not lead to p21*^Waf1/Cip1^* expression. These cells lack p16 and p14^ARF^ as well [57], and the lack of these two key regulators and the absence of active p53 impedes the ability of G55T2 cells to induce premature senescence. The importance of the p53/p21*^Waf1/Cip1^* regulatory axis for GC7 induced senescence becomes even more apparent after reintroduction of wild type p53 into G55T2 cells. These cells show a senescence phenotype when treated with GC7, whereas cells transduced with a control vector do not. This sheds more light on the connection between p53 and eIF-5A which has been established previously by Li *et al.* [59] as well as Rahman-Roblick *et al*. [60]. Rahman-Roblick *et al.* revealed an upregulation of eIF-5A expression after induction of p53-dependent cell death. *Li et al.* demonstrated an increase of p53 level (and expression of some of its target genes, like p21 and Mdm2) upon endogenous overexpression of eIF-5A. However, they did not confirm the hypusine status of the exogenous eIF-5A, which can remain largely unmodified if not complemented by an endogenous overexpression of DHS [55]. Thus, it remains unclear if an overexpression or the accumulation of non-hypusinated eIF-5A plays a role in p53-activation. Our results indicate the latter, since GC7-treated cells quickly accumulate non-modified eIF-5A. Our results highlight the role of p53 in eIF5A depend regulatory mechanisms. Further, we could demonstrate a connection of DHS inhibition, accumulation of non-modified eIF-5A, cell cycle arrest and induction of p53-dependent premature senescence. These cells show a senescence phenotype when treated with GC7, whereas cells transduced with a control vector do not. These observations underscore that inhibition of functional eIF-5A also regulates p53-independent processes leading to a decrease in proliferation and growth retardation. This might be of great impact for the treatment of p53-defective glioblastomas. Importantly, GC7 had no notable effect on proliferation, viability or morphology of astrocytes.

Since GC7 is a polyamine analogue, it probably affects other polyamine dependent cellular processes at a certain concentration. The use of non-spermidine related DHS inhibitors (e.g. CNI-1493) could help to circumvent these off-target effects [61]. To exclude such side effects as primary mode of GC7, we knocked down DHS by stable expression of a shRNA-pool against DHS mRNA in glioblastoma cells. DHS knockdown by a pool of four different shRNA resulted in a significant reduction of cell growth and a phenotype, similar to cells incubated with GC7. Knockdown of eIF-5A, the target of DHS mediated hypusine synthesis only yielded a moderately reduced proliferation. This contrast to cells with reduced DHS activity could be explained in two ways. (i) eIF-5A is highly expressed in GBM cell lines, thus the transduced shRNAs against its mRNA are not able to convey a knockdown to a level that is needed to achieve a more pronounced effect on the cells. However, this contrasts with our finding that GBM cells with moderate and high knockdown of eIF-5A show no difference in proliferation. (ii) Unmodified eIF-5A is still present in the cell when DHS is inhibited or knocked down. This form of eIF-5A was shown to have proapoptotic properties, which contribute to the antiproliferative effect of DHS inhibition [50], [62]. Although we could not observe apoptosis in our experiments, this may depend on experimental settings or tissue origin of cells. In addition we cannot exclude other antiproliferative effects of non-hypusinated eIF-5A.

Treatment of glioblastoma patients with DNA damaging agents such as BCNU or TMZ is the current standard of adjuvant therapy after surgical removal of GBM. Thus we were interested in the potential of DHS inhibition in enhancing TMZ/BCNU mediated antiproliferative effects. The G_2_/M arrest (resulting in senescence and/or apoptosis, depending on the experimental setup) induced by TMZ has been studied in the past [63], [64]. Our results suggest an additive effect of combined TMZ/BCNU and GC7 treatment, indicated by improved reduction in proliferation. This is also reflected by the cell cycle of treated cells, which show a combination of GC7 induced G_1_- and TMZ/BCNU characteristic G_2_/M-arrest and accumulation of >4n cells. Thus we speculate that the enhanced antiproliferative effect of a combination therapy is elicited by simultaneous cell cycle inhibition at G_1_/S phase and G_2_/M phase checkpoints. TMZ and BCNU have no effect on the hypusine status of eIF-5A, hence the combination of alkylating agents and DHS inhibitors would provide a two-tier approach in engaging GBM cells. Despite the involvement of eIF-5A in apoptotic processes in various cancer models, we could not confirm such role in GBM cells. Also in TMZ-stressed cells, we could not observe any proapoptotic effect after inhibition of hypusination, indicating that eIF-5A and its hypusine modification have a role in proliferation control rather than in regulation of apoptosis in GBM cells.

Taken together, these data establish an important role for eIF-5A and the hypusine synthesizing enzymes in proliferation of glioblastoma cells. We also showed, in a proof of principle approach that the inhibition the DHS enzyme might serve as an option to reduce growth of GBM cells. However, more in depth studies and the design of DHS inhibitors, which are more suitable for in vivo applications, are needed. Although eIF-5A, DHS and DOHH are highly conserved and likewise expressed in healthy tissues, their overexpression in aggressive astrocytomas and the enhanced responsiveness of GMB cells compared to normal astrocytes render these protein potential therapeutic targets in this intracranial neoplasm. Further, eIF-5A and the hypusine synthesis pathway are highly conserved in eukaryotes [65]. This eventually reduces the probability of tumor cells to circumvent the inhibition of hypusine synthesis.

## Supporting Information

Figure S1 **Schematic representation of the lentiviral vector expressing human wild type p53 and control vector, drawn as integrated provirus.** A PCR fragment of p53 cDNA has been cloned into the multiple cloning site of LeGO-iCer2-Puro+ (EcoRI and NotI) and verified by sequencing. Vector elements (not drawn to scale): SIN-LTR, self-inactivating long terminal repeat; Ψ, packaging signal; RRE, rev-responsive element; cPPT, central polypurine tract; SFFV, Spleen focus-forming virus enhancer/promoter; wt p53, cDNA coding for human wild-type p53; IRES, internal ribosome entry site of the Encephalo myocarditis virus; Cerulean, a cyan fluorescent protein; 2A, self cleaving peptide of Porcine Teschovirus-1 (P2A); PuroR, codon optimized cDNA of puromycin N-acetyltransferase (puromycin resistance); wPRE, Woodchuck hepatitis virus post-transcriptional regulatory element.
(DOC)

Figure S2 **Kaplan–Meier survival plots show the survival of glioma patients with differential expression of eIF-5A.** Patiens with upregulation of eIF-5A (red line; *n = *82) and patients with intermediate expression (yellow; *n = *241) are shown. Log-rank *p* value (upregulated vs. intermediate, mean of all reporters): 0.0433240981. Data obtained from National Cancer Institute REMBRANDT database (https://caintegrator.nci.nih.gov/rembrandt).
(DOC)

Table S1 **Raw data of the immunostained TMAs.** TMAs were stained with anti-eIF-5A, -A2, DHS and DOHH antibodies. Staining intensities were quantified in 4 grades (0: none, 1: slight staining in up to 20% of cells, 2: moderate or strong staining in up to 50% of cells, 3: moderate to strong staining of >50% of cells) and only tumor cells were assessed. Tumortypes are 1 = astrocytomas and 2 = oligodendrogliomas. Grade represents the WHO glioma grade. Sex is coded as follows: 1 = female; 2 = male. Age is given in years. Localisation is coded: 1 = frontal lobe; 2 = temporal lobe; 3 = central; 4 = occipital cortex; 5 = cerebellum; 6 = spinal cord; 7 = opticus.
(DOC)

Table S2 **Mean staining intensity of DHS and DOHH immunolabelled TMAs with SEM.**
(DOC)
