# Supplementary material for: Expression of Eukaryotic Initiation Factor 5A and Hypusine Forming Enzymes in Glioblastoma Patient Samples: Implications for New Targeted Therapies
Source: PLoS One. 2012 Aug 21;7(8):e43468. doi: 10.1371/journal.pone.0043468 (PMC3424167; doi:10.1371/journal.pone.0043468)
Supplement: Figure S1 — Schematic representation of the lentiviral vector expressing human wild type p53 and control vector, drawn as integrated provirus. A PCR fragment of p53 cDNA has been cloned into the multiple cloning site of LeGO-iCer2-Puro+ (EcoRI and NotI) and verified by sequencing. Vector elements (not drawn to scale): SIN-LTR, self-inactivating long terminal repeat; Ψ, packaging signal; RRE, rev-responsive element; cPPT, central polypurine tract; SFFV, Spleen focus-forming virus enhancer/promoter; wt p53, cDNA coding for human wild-type p53; IRES, internal ribosome entry site of the Encephalo myocarditis virus; Cerulean, a cyan fluorescent protein; 2A, self cleaving peptide of Porcine Teschovirus-1 (P2A); PuroR, codon optimized cDNA of puromycin N-acetyltransferase (puromycin resistance); wPRE, Woodchuck hepatitis virus post-transcriptional regulatory element. (DOC) [file pone.0043468.s001.doc]

**LeGO-iCer2-Puro^+^-p53**

**wPRE**

**SFFV**

**Cerulean**

**Ψ cPPT**

**Δ LTR**

**RRE**

**Δ LTR**

**Puro^R^**

**IRES**

**wt p53**

**2A**

**LeGO-Cer2-Puro^+^**

**wPRE**

**SFFV**

**Cerulean**

**Ψ cPPT**

**Δ LTR**

**RRE**

**Δ LTR**

**Puro^R^**

**2A**

**Supplementary Figure S1:** Schematic representation of the lentiviral vector expressing human wild type p53, drawn as integrated provirus. A PCR fragment of p53 cDNA has been cloned into the multiple cloning site of LeGO-iCer2-Puro+ (EcoRI and NotI) and verified by sequencing.

Vector elements (not drawn to scale): SIN-LTR, self-inactivating long terminal repeat; Ψ, packaging signal; RRE, rev-responsive element; cPPT, central polypurine tract; SFFV, Spleen focus-forming virus enhancer/promoter; wt p53, cDNA coding for human wild-type p53; IRES, internal ribosome entry site of the Encephalo myocarditis virus; Cerulean, a cyan fluorescent protein; 2A, self cleaving peptide of Porcine Teschovirus-1 (P2A); PuroR, codon optimized cDNA of puromycin N-acetyltransferase (puromycin resistance); wPRE, Woodchuck hepatitis virus post-transcriptional regulatory element.
