# Supplementary material for: Expression of Eukaryotic Initiation Factor 5A and Hypusine Forming Enzymes in Glioblastoma Patient Samples: Implications for New Targeted Therapies
Source: PLoS One. 2012 Aug 21;7(8):e43468. doi: 10.1371/journal.pone.0043468 (PMC3424167; doi:10.1371/journal.pone.0043468)
Supplement: Figure S2 — Kaplan–Meier survival plots show the survival of glioma patients with differential expression of eIF-5A. Patiens with upregulation of eIF-5A (red line; n = 82) and patients with intermediate expression (yellow; n = 241) are shown. Log-rank p value (upregulated vs. intermediate, mean of all reporters): 0.0433240981. Data obtained from National Cancer Institute REMBRANDT database (https://caintegrator.nci.nih.gov/rembrandt). (DOC) [file pone.0043468.s002.doc]

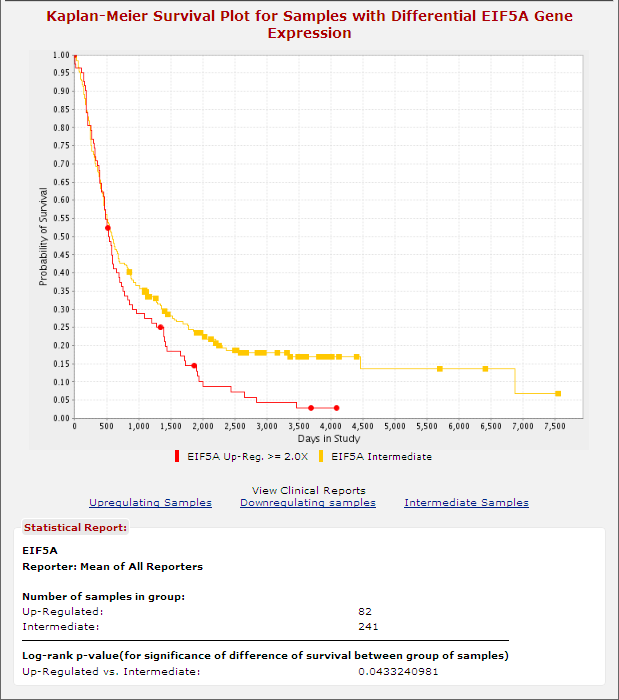


**Supplementary Figure S2:** Kaplan–Meier survival plots show the survival of glioma patients with differential expression of eIF-5A. Patiens with upregulation of eIF-5A (red line; *n* = 82) and patients with intermediate expression (yellow; *n* = 241) are shown. Log-rank *p* value (upregulated vs. intermediate, mean of all reporters): 0.0433240981. Data obtained from National Cancer Institute REMBRANDT database (https://caintegrator.nci.nih.gov/rembrandt).
