# Supplementary material for: Expression of Eukaryotic Initiation Factor 5A and Hypusine Forming Enzymes in Glioblastoma Patient Samples: Implications for New Targeted Therapies
Source: PLoS One. 2012 Aug 21;7(8):e43468. doi: 10.1371/journal.pone.0043468 (PMC3424167; doi:10.1371/journal.pone.0043468)
Supplement: Table S1 — Raw data of the immunostained TMAs. TMAs were stained with anti-eIF-5A, -A2, DHS and DOHH antibodies. Staining intensities were quantified in 4 grades (0: none, 1: slight staining in up to 20% of cells, 2: moderate or strong staining in up to 50% of cells, 3: moderate to strong staining of >50% of cells) and only tumor cells were assessed. Tumortypes are 1 = astrocytomas and 2 = oligodendrogliomas. Grade represents the WHO glioma grade. Sex is coded as follows: 1 = female; 2 = male. Age is given in years. Localisation is coded: 1 = frontal lobe; 2 = temporal lobe; 3 = central; 4 = occipital cortex; 5 = cerebellum; 6 = spinal cord; 7 = opticus. (DOC) [file pone.0043468.s003.doc]

**Supplementary Table S1:** Raw data of the immunostained TMAs. TMAs were stained with anti-eIF-5A, -A2, DHS and DOHH antibodies. Staining intensities were quantified in 4 grades (0: none, 1: slight staining in up to 20% of cells, 2: moderate or strong staining in up to 50% of cells, 3: moderate to strong staining of >50% of cells) and only tumor cells were assessed. Tumortypes are 1 = astrocytomas and 2 = oligodendrogliomas. Grade represents the WHO glioma grade. Sex is coded as follows: 1 = female; 2 = male. Age is given in years. Localisation is coded: 1 = frontal lobe; 2 = temporal lobe; 3 = central; 4 = occipital cortex; 5 = cerebellum; 6 = spinal cord; 7 = opticus.

| **Sample** | **DHS** | **elF5A2** | **elF5A** | **DOHH** | **Tumortype** | **Grade** | **Sex** | **Age** | **Localisation** |
| --- | --- | --- | --- | --- | --- | --- | --- | --- | --- |
| A1 1-2 | 1.00 | 0.00 | 3.00 | 1.00 | 1.00 | 1.00 | 1.00 | 6.00 |  |
| A1 3-4 | 0.00 | 0.00 | 2.50 | 2.00 | 1.00 | 1.00 | 2.00 | 30.00 | 7.00 |
| A1 5-6 | 0.00 | 0.00 | 2.00 | 1.00 | 1.00 | 1.00 | 2.00 | 15.00 | 7.00 |
| A1 7-8 | 0.00 | 0.00 | 2.00 | 2.00 | 1.00 | 1.00 | 2.00 | 20.00 | 5.00 |
| A1 9-10 | 0.00 | 0.00 | 3.00 | 2.00 | 1.00 | 1.00 | 1.00 | 25.00 | 1.00 |
| A2 1-2 | 0.00 | 0.00 | 1.00 | 1.00 | 1.00 | 1.00 | 2.00 | 3.00 | 5.00 |
| A2 3-4 | 1.00 | 0.00 | 2.00 | 1.00 | 1.00 | 1.00 | 2.00 | 14.00 | 6.00 |
| A2 5-6 | 0.00 | 0.00 | 2.00 | 2.00 | 1.00 | 1.00 | 2.00 | 13.00 | 5.00 |
| A2 7-8 | 0.00 | 0.00 | 2.00 | 2.00 | 1.00 | 1.00 | 2.00 | 12.00 |  |
| A2 9-10 | 0.00 | 0.00 | 2.00 | 2.00 | 1.00 | 1.00 | 1.00 | 12.00 | 5.00 |
| A3 1-2 | 0.00 | 1.00 | 2.00 | 2.00 | 1.00 | 1.00 | 1.00 | 28.00 | 6.00 |
| A3 3-4 | 0.00 | 0.00 | 1.00 | 0.00 | 1.00 | 1.00 | 1.00 | 15.00 | 6.00 |
| A3 5-6 | 1.00 | 0.00 | 2.00 | 3.00 | 1.00 | 1.00 | 2.00 | 4.00 | 5.00 |
| A3 7-8 | 0.00 | 0.00 | 2.00 | 1.00 | 1.00 | 1.00 | 2.00 | 8.00 | 7.00 |
| A3 9-10 | 0.00 | 0.00 | 3.00 | 1.00 | 1.00 | 1.00 | 1.00 | 16.00 | 5.00 |
| A4 1-2 | 1.00 | 0.00 | 3.00 | 2.00 | 1.00 | 1.00 | 1.00 | 6.00 | 7.00 |
| A4 3-4 | 0.00 | 0.00 | 0.00 | 0.00 | 1.00 | 1.00 | 1.00 | 12.00 | 5.00 |
| A4 5-6 | 0.00 | 0.00 | 1.00 | 0.50 | 1.00 | 1.00 | 1.00 | 16.00 | 5.00 |
| A4 7-8 | 0.50 | 0.00 | 2.00 | 1.00 | 1.00 | 1.00 | 1.00 | 6.00 | 5.00 |
| A4 9-10 | 0.50 | 0.00 | 0.50 | 0.00 | 1.00 | 1.00 | 2.00 | 22.00 | 5.00 |
| A5 1-2 | 2.00 | 0.00 | 2.00 | 1.00 | 1.00 | 1.00 | 1.00 | 15.00 | 7.00 |
| A5 3-4 | 0.00 | 0.00 | 2.00 | 1.00 | 1.00 | 1.00 | 1.00 | 28.00 | 5.00 |
| A5 5-6 | 0.00 | 0.00 | 2.00 | 2.00 | 1.00 | 1.00 | 2.00 | 13.00 | 5.00 |
| A5 7-8 | 0.00 | 0.00 | 3.00 | 2.00 | 1.00 | 1.00 | 2.00 | 14.00 | 5.00 |
| A5 9-10 | 0.00 | 0.00 | 0.00 | 1.00 | 1.00 | 1.00 | 1.00 | 13.00 | 5.00 |
| A6 1-2 | 1.00 | 0.00 | 1.00 | 0.00 | 1.00 | 1.00 | 1.00 | 8.00 | 5.00 |
| A6 3-4 | 0.00 | 0.00 | 2.00 | 1.00 | 1.00 | 1.00 | 1.00 | 17.00 | 5.00 |
| A6 5-6 | 1.00 | 0.00 | 2.00 | 1.00 | 1.00 | 1.00 | 2.00 | 33.00 | 1.00 |
| A6 7-8 | 0.50 | 0.00 | 2.50 | 1.00 | 1.00 | 1.00 | 1.00 | 2.00 | 6.00 |
| A6 9-10 | 0.00 | 0.00 | 2.00 | 1.00 | 1.00 | 1.00 | 2.00 | 12.00 | 5.00 |
| A7 1-2 | 0.00 | 0.00 | 3.00 | 1.00 | 1.00 | 2.00 | 2.00 | 31.00 | 3.00 |
| A7 3-4 | 0.00 | 0.00 | 2.00 | 2.00 | 1.00 | 2.00 | 1.00 | 43.00 | 2.00 |
| A7 5-6 | 0.00 | 0.00 | 2.00 | 0.00 | 1.00 | 2.00 | 2.00 | 40.00 | 2.00 |
| A7 7-8 | 0.00 | 0.00 | 2.00 | 1.00 | 1.00 | 2.00 | 2.00 | 40.00 | 2.00 |
| A7 9-10 | 0.00 | 0.00 | 2.00 | 1.00 | 1.00 | 2.00 | 2.00 | 28.00 | 1.00 |
| A8 1-2 | 0.00 | 0.00 | 3.00 | 1.00 | 1.00 | 2.00 | 2.00 | 40.00 | 2.00 |
| A8 3-4 | 0.00 | 0.00 | 3.00 | 1.00 | 1.00 | 2.00 | 2.00 | 15.00 | 5.00 |
| A8 5-6 | 1.00 | 0.00 | 2.00 | 1.00 | 1.00 | 2.00 | 2.00 | 58.00 | 2.00 |
| A8 7-8 | 0.00 | 0.00 | 2.00 | 1.00 | 1.00 | 2.00 | 2.00 | 18.00 | 1.00 |
| A8 9-10 | 0.50 | 0.00 | 2.00 | 0.50 | 1.00 | 2.00 | 1.00 | 46.00 | 1.00 |
| A9 1-2 | 1.50 | 0.00 | 1.00 | 0.00 | 1.00 | 2.00 | 2.00 | 43.00 | 4.00 |
| A9 3-4 | 1.00 | 0.00 | 2.00 | 1.00 | 1.00 | 2.00 | 1.00 | 33.00 |  |
| A9 5-6 | 0.00 | 0.00 | 2.00 | 2.00 | 1.00 | 2.00 | 1.00 | 22.00 | 2.00 |
| A9 7-8 | 1.00 | 0.00 | 1.00 | 1.00 | 1.00 | 2.00 | 2.00 | 24.00 | 1.00 |
| A9 9-10 | 0.50 | 0.00 | 1.00 | 0.00 | 1.00 | 2.00 | 1.00 | 52.00 | 1.00 |
| A10 1-2 | 0.00 | 0.00 | 3.00 | 1.00 | 1.00 | 2.00 | 2.00 | 57.00 | 1.00 |
| A10 3-4 | 0.00 | 0.00 | 1.00 | 0.00 | 1.00 | 2.00 | 2.00 | 52.00 | 1.00 |
| A10 5-6 | 0.00 | 0.00 | 0.00 | 2.00 | 1.00 | 2.00 | 2.00 | 44.00 | 1.00 |
| A10 7-8 | 0.00 | 0.00 | 2.00 | 2.00 | 1.00 | 2.00 | 2.00 | 43.00 |  |
| A10 9-10 | 0.00 | 0.00 | 3.00 | 2.00 | 1.00 | 2.00 | 2.00 | 33.00 |  |
| A11 1-2 | 1.00 | 0.00 | 3.00 | 1.00 | 1.00 | 2.00 | 1.00 | 51.00 | 1.00 |
| A11 3-4 | 0.00 | 0.00 | 22.00 | 1.00 | 1.00 | 2.00 | 2.00 | 37.00 | 2.00 |
| A11 5-6 | 0.00 | 0.00 | 3.00 | 3.00 | 1.00 | 2.00 | 1.00 | 78.00 | 1.00 |
| A11 7-8 | 0.00 | 0.00 | 2.00 | 1.00 | 1.00 | 2.00 | 1.00 | 67.00 | 2.00 |
| A11 9-10 | 0.00 | 0.00 | 0.00 | 0.00 | 1.00 | 2.00 | 1.00 | 56.00 | 1.00 |
| A12 1-2 | 1.00 | 0.00 | 2.00 | 1.00 | 1.00 | 2.00 | 2.00 | 39.00 | 3.00 |
| A12 3-4 | 0.00 | 0.00 | 2.00 | 1.00 | 1.00 | 2.00 | 2.00 | 39.00 |  |
| A12 5-6 | 0.00 | 0.00 | 2.00 | 2.00 | 1.00 | 2.00 | 2.00 | 38.00 | 2.00 |
| A12 7-8 | 0.00 | 0.00 | 2.00 | 1.00 | 1.00 | 2.00 | 2.00 | 30.00 | 4.00 |
| A12 9-10 | 0.00 | 0.00 | 1.00 | 2.00 | 1.00 | 2.00 | 2.00 | 52.00 | 2.00 |
| A13 1-2 | 0.00 | 0.00 | 2.00 | 1.00 | 1.00 | 3.00 | 2.00 | 46.00 | 1.00 |
| A13 3-4 | 0.00 | 0.00 | 3.00 | 2.00 | 1.00 | 3.00 | 2.00 | 42.00 | 1.00 |
| A13 5-6 | 0.00 | 0.00 | 3.00 | 3.00 | 1.00 | 3.00 | 2.00 | 44.00 | 2.00 |
| A13 7-8 | 0.00 | 0.00 | 1.00 | 2.00 | 1.00 | 3.00 | 1.00 | 40.00 | 3.00 |
| A13 9-10 | 0.00 | 0.00 | 2.00 | 2.00 | 1.00 | 3.00 | 2.00 | 32.00 | 2.00 |
| A14 1-2 | 1.50 | 0.00 | 2.00 | 2.00 | 1.00 | 3.00 | 1.00 | 30.00 | 1.00 |
| A14 3-4 | 1.00 | 0.00 | 3.00 |  | 1.00 | 3.00 | 2.00 | 29.00 | 3.00 |
| A14 5-6 | 0.00 | 0.00 | 2.00 | 2.00 | 1.00 | 3.00 | 2.00 | 26.00 | 1.00 |
| A14 7-8 | 0.00 | 0.00 | 2.00 | 2.00 | 1.00 | 3.00 | 2.00 | 36.00 |  |
| A14 9-10 | 0.00 | 0.00 | 1.00 | 1.00 | 1.00 | 3.00 | 2.00 | 51.00 | 1.00 |
| A15 1-2 | 0.50 | 0.00 | 3.00 | 3.00 | 1.00 | 3.00 | 2.00 | 54.00 | 1.00 |
| A15 3-4 | 0.00 | 0.00 | 3.00 | 2.00 | 1.00 | 3.00 | 2.00 | 60.00 | 1.00 |
| A15 5-6 | 0.00 | 0.00 | 1.00 | 1.00 | 1.00 | 3.00 | 2.00 | 67.00 |  |
| A15 7-8 | 0.00 | 0.00 | 1.00 | 1.00 | 1.00 | 3.00 | 1.00 | 68.00 | 3.00 |
| A15 9-10 | 0.00 | 0.00 | 1.00 | 1.00 | 1.00 | 3.00 | 2.00 | 49.00 | 1.00 |
| A16 1-2 | 0.00 | 0.00 | 2.00 | 1.00 | 1.00 | 3.00 | 1.00 | 33.00 | 1.00 |
| A16 3-4 | 0.00 | 0.00 | 3.00 | 2.00 | 1.00 | 3.00 | 2.00 | 55.00 |  |
| A16 5-6 | 0.00 | 0.00 | 3.00 | 1.00 | 1.00 | 3.00 | 1.00 | 25.00 | 2.00 |
| A16 7-8 | 0.00 | 0.00 | 2.00 | 1.00 | 1.00 | 3.00 | 2.00 | 71.00 | 1.00 |
| A16 9-10 | 0.00 | 0.00 | 2.00 | 1.00 | 1.00 | 3.00 | 1.00 | 50.00 | 2.00 |
| A17 1-2 | 0.00 | 0.00 | 2.00 | 1.00 | 1.00 | 3.00 | 2.00 | 39.00 | 2.00 |
| A17 3-4 | 0.00 | 0.00 | 2.00 | 1.00 | 1.00 | 3.00 | 1.00 | 16.00 | 3.00 |
| A17 5-6 | 1.00 | 0.00 |  | 1.00 | 1.00 | 3.00 | 2.00 | 52.00 | 1.00 |
| A17 7-8 |  | 0.00 | 2.00 | 1.00 | 1.00 | 3.00 | 2.00 | 39.00 | 2.00 |
| A17 9-10 | 0.00 | 0.00 | 2.00 | 2.00 | 1.00 | 3.00 | 1.00 | 43.00 |  |
| A18 1-2 | 0.00 | 0.00 | 1.00 | 2.00 | 1.00 | 3.00 | 2.00 | 63.00 |  |
| A18 3-4 | 0.00 | 0.00 | 2.00 | 1.00 | 1.00 | 3.00 | 2.00 | 42.00 | 1.00 |
| B1 1-2 | 1.00 | 0.00 | 2.00 | 3.00 | 1.00 | 4.00 | 2.00 | 74.00 | 3.00 |
| B1 3-4 | 0.00 | 0.00 | 1.00 | 3.00 | 1.00 | 4.00 | 2.00 | 51.00 | 2.00 |
| B1 5-6 | 0.50 | 0.00 | 2.00 | 2.00 | 1.00 | 4.00 | 1.00 | 68.00 | 1.00 |
| B1 7-8 | 0.00 | 0.00 | 2.00 | 2.00 | 1.00 | 4.00 | 1.00 | 66.00 | 2.00 |
| B1 9-10 | 1.00 | 0.00 | 2.00 | 2.00 | 1.00 | 4.00 | 2.00 | 72.00 | 2.00 |
| B2 1-2 | 0.00 | 0.00 | 2.00 | 2.00 | 1.00 | 4.00 | 2.00 | 57.00 | 4.00 |
| B2 3-4 | 0.00 | 0.00 | 2.00 | 1.00 | 1.00 | 4.00 | 1.00 | 48.00 | 3.00 |
| B2 5-6 | 0.00 | 0.00 | 2.00 | 1.00 | 1.00 | 4.00 | 1.00 | 59.00 | 1.00 |
| B2 7-8 | 1.00 | 0.00 | 2.00 | 0.00 | 1.00 | 4.00 | 2.00 | 63.00 | 1.00 |
| B2 9-10 | 1.00 | 0.00 | 3.00 | 3.00 | 1.00 | 4.00 | 1.00 | 62.00 | 2.00 |
| B3 1-2 | 2.00 | 0.00 | 3.00 | 3.00 | 1.00 | 4.00 | 1.00 | 50.00 | 1.00 |
| B3 3-4 | 1.00 | 0.00 | 2.00 | 3.00 | 1.00 | 4.00 | 1.00 | 45.00 | 2.00 |
| B3 5-6 | 2.00 | 0.00 | 2.00 | 1.00 | 1.00 | 4.00 | 2.00 | 55.00 | 2.00 |
| B3 7-8 | 2.00 | 0.00 | 3.00 | 3.00 | 1.00 | 4.00 | 1.00 | 44.00 | 1.00 |
| B3 9-10 | 1.00 | 0.00 | 2.00 | 3.00 | 1.00 | 4.00 | 2.00 | 70.00 | 4.00 |
| B4 1-2 | 1.50 | 0.00 | 2.00 | 1.50 | 1.00 | 4.00 | 2.00 | 70.00 | 3.00 |
| B4 3-4 | 1.00 | 0.00 | 0.50 | 0.00 | 1.00 | 4.00 | 2.00 | 67.00 | 1.00 |
| B4 5-6 | 1.00 | 0.00 | 2.00 | 0.00 | 1.00 | 4.00 | 2.00 | 66.00 | 1.00 |
| B4 7-8 | 1.00 | 0.00 | 1.50 | 1.50 | 1.00 | 4.00 | 1.00 | 64.00 | 3.00 |
| B4 9-10 | 0.00 | 0.00 | 2.00 | 1.00 | 1.00 | 4.00 | 2.00 | 57.00 | 1.00 |
| B5 1-2 | 0.50 | 0.00 | 0.50 | 2.00 | 1.00 | 4.00 | 1.00 | 61.00 | 3.00 |
| B5 3-4 | 1.00 | 0.00 | 2.00 | 2.00 | 1.00 | 4.00 | 2.00 | 71.00 |  |
| B5 5-6 | 0.50 | 0.00 | 2.00 | 2.00 | 1.00 | 4.00 | 1.00 | 76.00 |  |
| B5 7-8 | 2.00 | 0.00 | 1.00 | 0.00 | 1.00 | 4.00 | 1.00 | 61.00 | 2.00 |
| B5 9-10 | 1.00 | 0.00 | 2.00 | 3.00 | 1.00 | 4.00 | 2.00 | 72.00 | 3.00 |
| B6 1-2 | 1.00 | 0.00 | 1.50 | 0.00 | 1.00 | 4.00 | 2.00 | 67.00 |  |
| B6 3-4 | 0.50 | 0.00 | 2.00 | 2.00 | 1.00 | 4.00 | 2.00 | 74.00 | 1.00 |
| B6 5-6 | 2.00 | 0.00 | 1.50 | 1.00 | 1.00 | 4.00 | 1.00 | 57.00 | 4.00 |
| B6 7-8 | 2.00 | 0.00 | 1.50 | 3.00 | 1.00 | 4.00 | 1.00 | 38.00 |  |
| B6 9-10 | 0.00 | 0.00 | 2.00 | 1.00 | 1.00 | 4.00 | 2.00 | 66.00 |  |
| B7 1-2 | 0.00 | 0.00 | 2.00 | 1.00 | 2.00 | 2.00 | 2.00 | 40.00 |  |
| B7 3-4 | 0.00 | 0.00 | 3.00 | 3.00 | 2.00 | 2.00 | 1.00 | 17.00 |  |
| B7 5-6 | 0.00 | 0.00 | 2.00 | 1.00 | 2.00 | 2.00 | 2.00 | 35.00 |  |
| B7 7-8 | 0.00 | 0.00 | 3.00 | 3.00 | 2.00 | 2.00 | 1.00 | 38.00 |  |
| B7 9-10 | 0.00 | 0.00 | 2.00 | 1.00 | 2.00 | 2.00 | 1.00 | 48.00 | 2.00 |
| B8 1-2 | 0.00 | 0.00 | 2.00 | 2.00 | 2.00 | 2.00 | 2.00 | 33.00 | 1.00 |
| B8 3-4 | 0.00 | 0.00 | 2.00 | 2.00 | 2.00 | 2.00 | 2.00 | 44.00 | 4.00 |
| B8 5-6 | 0.00 | 0.00 | 1.50 | 1.00 | 2.00 | 2.00 | 2.00 | 56.00 | 2.00 |
| B8 7-8 | 0.00 | 0.00 | 1.00 | 1.00 | 2.00 | 2.00 | 1.00 | 48.00 | 2.00 |
| B8 9-10 | 1.00 | 0.00 | 2.00 | 2.00 | 2.00 | 2.00 | 1.00 | 49.00 | 1.00 |
| B9 1-2 | 1.00 | 0.00 | 2.00 | 3.00 | 2.00 | 2.00 | 2.00 | 33.00 | 1.00 |
| B9 3-4 | 1.00 | 0.00 | 0.00 | 0.00 | 2.00 | 2.00 | 1.00 | 30.00 | 1.00 |
| B9 5-6 | 0.00 | 0.00 | 2.00 | 1.00 | 2.00 | 2.00 | 2.00 | 48.00 | 1.00 |
| B9 7-8 | 0.00 | 0.00 | 2.00 | 1.00 | 2.00 | 2.00 | 2.00 | 59.00 | 1.00 |
| B9 9-10 | 0.00 | 0.00 | 2.00 | 1.00 | 2.00 | 2.00 | 1.00 | 53.00 | 2.00 |
| B10 1-2 | 0.00 | 0.00 | 2.00 | 1.00 | 2.00 | 2.00 | 1.00 | 28.00 | 1.00 |
| B10 3-4 | 0.00 | 0.00 | 1.00 | 1.00 | 2.00 | 2.00 | 1.00 | 44.00 | 1.00 |
| B10 5-6 | 0.50 | 0.00 | 1.00 | 1.00 | 2.00 | 2.00 | 1.00 | 50.00 | 1.00 |
| B10 7-8 | 0.00 | 0.00 | 2.00 | 2.00 | 2.00 | 2.00 | 2.00 | 41.00 | 1.00 |
| B10 9-10 | 0.00 | 0.00 | 2.00 | 1.00 | 2.00 | 2.00 | 1.00 | 46.00 | 4.00 |
| B11 1-2 | 0.00 | 0.00 | 1.00 | 0.00 | 2.00 | 2.00 | 2.00 | 33.00 | 3.00 |
| B11 3-4 | 1.00 | 0.00 | 1.00 | 0.00 | 2.00 | 2.00 | 2.00 | 32.00 | 1.00 |
| B11 5-6 | 0.00 | 0.00 | 1.00 | 1.00 | 2.00 | 2.00 | 1.00 | 47.00 | 3.00 |
| B11 7-8 | 0.00 | 0.00 | 1.00 | 1.00 | 2.00 | 2.00 | 1.00 | 44.00 | 1.00 |
| B11 9-10 | 0.00 | 0.00 | 1.50 | 1.00 | 2.00 | 2.00 | 2.00 | 48.00 | 2.00 |
| B12 1-2 | 1.00 | 0.00 | 2.00 | 1.50 | 2.00 | 2.00 | 2.00 | 41.00 | 1.00 |
| B12 3-4 | 1.50 | 0.00 | 1.00 | 1.00 | 2.00 | 2.00 | 2.00 | 35.00 |  |
| B12 5-6 | 0.50 | 0.00 | 2.00 | 2.00 | 2.00 | 2.00 | 1.00 | 36.00 |  |
| A13 1-2 | 0.00 | 0.00 | 2.00 | 2.00 | 2.00 | 3.00 | 2.00 | 34.00 | 1.00 |
| B13 3-4 | 0.00 | 0.00 | 2.00 | 2.50 | 2.00 | 3.00 | 1.00 | 33.00 | 1.00 |
| B13 5-6 | 1.00 | 0.00 | 3.00 | 3.00 | 2.00 | 3.00 | 2.00 | 34.00 | 2.00 |
| B13 7-8 | 1.50 | 0.00 | 3.00 | 2.00 | 2.00 | 3.00 | 1.00 | 48.00 | 1.00 |
| B13 9-10 | 0.50 | 0.00 | 3.00 | 3.00 | 2.00 | 3.00 | 2.00 | 41.00 | 1.00 |
| B14 1-2 | 0.00 | 0.00 | 1.00 | 1.00 | 2.00 | 3.00 | 2.00 | 37.00 | 3.00 |
| B14 3-4 | 1.00 | 0.00 | 2.00 | 2.00 | 2.00 | 3.00 | 2.00 | 36.00 | 2.00 |
| A14 5-6 | 1.00 | 0.00 | 2.00 | 2.00 | 2.00 | 3.00 | 1.00 | 56.00 | 1.00 |
| B14 7-8 | 0.00 | 0.00 | 2.00 | 2.00 | 2.00 | 3.00 | 2.00 | 47.00 | 1.00 |
| B14 9-10 | 1.00 | 0.00 | 2.00 | 3.00 | 2.00 | 3.00 | 1.00 | 34.00 | 3.00 |
| B15 1-2 | 0.50 | 0.00 | 2.00 | 3.00 | 2.00 | 3.00 | 1.00 | 50.00 | 1.00 |
| B15 3-4 | 0.00 | 0.00 | 2.00 | 1.00 | 2.00 | 3.00 | 1.00 | 46.00 | 1.00 |
| B15 5-6 | 1.00 | 0.00 | 2.00 | 1.00 | 2.00 | 3.00 | 2.00 | 13.00 | 1.00 |
| B15 7-8 | 2.00 | 0.00 | 3.00 | 2.00 | 2.00 | 3.00 | 2.00 | 59.00 | 2.00 |
| B15 9-10 | 0.00 | 0.00 | 1.00 | 1.00 | 2.00 | 3.00 | 2.00 | 57.00 | 1.00 |
| B16 1-2 | 0.00 | 0.00 | 2.00 | 0.00 | 2.00 | 3.00 | 1.00 | 61.00 | 2.00 |
| B16 3-4 | 0.00 | 0.00 | 2.00 | 0.00 | 2.00 | 3.00 | 1.00 | 53.00 | 1.00 |
| B16 5-6 | 1.00 | 0.00 | 2.00 | 3.00 | 2.00 | 3.00 | 2.00 | 49.00 | 2.00 |
| B16 7-8 | 1.00 | 0.00 | 1.00 | 0.00 | 2.00 | 3.00 | 2.00 | 58.00 | 1.00 |
| B16 9-10 | 0.00 | 0.00 | 1.00 | 0.00 | 2.00 | 3.00 | 2.00 | 57.00 | 2.00 |
| B17 1-2 | 1.00 | 0.00 | 1.00 | 1.00 | 2.00 | 3.00 | 2.00 | 30.00 | 1.00 |
| B17 3-4 | 1.00 | 0.00 | 2.00 | 1.00 | 2.00 | 3.00 | 2.00 | 34.00 | 1.00 |
| B17 5-6 | 2.00 | 0.00 | 2.00 | 1.50 | 2.00 | 3.00 | 1.00 | 66.00 | 1.00 |
| B17 7-8 | 1.00 | 0.00 | 2.00 | 2.00 | 2.00 | 3.00 | 1.00 | 60.00 | 1.00 |
| B17 9-10 | 1.00 | 0.00 | 2.00 | 1.00 | 2.00 | 3.00 | 1.00 | 42.00 | 1.00 |
| B18 1-2 | 0.00 | 0.00 | 2.00 | 0.00 | 2.00 | 3.00 | 2.00 | 40.00 |  |
| B18 3-4 | 0.00 | 0.00 | 2.00 | 0.00 | 2.00 | 3.00 | 1.00 | 50.00 |  |
| B18 5-6 | 0.00 | 0.00 | 1.00 | 0.00 | 2.00 | 3.00 | 1.00 | 65.00 | 1.00 |
