# Supplementary material for: Expression of Eukaryotic Initiation Factor 5A and Hypusine Forming Enzymes in Glioblastoma Patient Samples: Implications for New Targeted Therapies
Source: PLoS One. 2012 Aug 21;7(8):e43468. doi: 10.1371/journal.pone.0043468 (PMC3424167; doi:10.1371/journal.pone.0043468)
Supplement: Table S2 — Mean staining intensity of DHS and DOHH immunolabelled TMAs with SEM. (DOC) [file pone.0043468.s004.doc]

Supplementary Table S2: Mean staining intensity of DHS and DOHH immunolabelled TMAs with SEM.

| **WHO grade** | **DHS** | **DHS SEM** | **DOHH** | **DOHH SEM** |
| --- | --- | --- | --- | --- |
| 1 | 0.3167 | 0.1015 | 1.25 | 0.135 |
| 2 | 0.2632 | 0.07046 | 1.2069 | 0.1015 |
| 3 | 0.3909 | 0.08514 | 1.4545 | 0.1241 |
| 4 | 0.9167 | 0.1269 | 1.6333 | 0.1958 |
